# Genome-Wide Identification and Classification of Arabinogalactan Proteins Gene Family in *Gossypium* Species and *GhAGP50* Increases Numbers of Epidermal Hairs in *Arabidopsis*

**DOI:** 10.3390/ijms26094159

**Published:** 2025-04-27

**Authors:** Renhui Wei, Ziru Guo, Zheng Yang, Yanpeng Zhao, Haoliang Yan, Muhammad Tehseen Azhar, Yamin Zhang, Gangling Li, Jingtao Pan, Aiying Liu, Wankui Gong, Qun Ge, Juwu Gong, Youlu Yuan, Haihong Shang

**Affiliations:** 1 State Key Laboratory of Cotton Bio-Breeding and Integrated Utilization, Institute of Cotton Research, Chinese Academy of Agricultural Sciences, Anyang 455000, China; wrh7117@163.com (R.W.); 18838990553@163.com (Z.G.); yanhl1989@163.com (H.Y.); panjingtao@caas.cn (J.P.); liuaiying@caas.cn (A.L.); gongwankui@caas.cn (W.G.); gequn@caas.cn (Q.G.); gongjuwu@caas.cn (J.G.); 2 Zhengzhou Research Base, State Key Laboratory of Cotton Bio-Breeding and Integrated Utilization, School of Agricultural Sciences, Zhengzhou University, Zhengzhou 450001, China; yangz@zzu.edu.cn (Z.Y.); ypzhao@zzu.edu.cn (Y.Z.); tehseenpbg@uaf.edu.pk (M.T.A.); ymzhang2017@163.com (Y.Z.); ligangling@zzu.edu.cn (G.L.); 3 Department of Plant Breeding and Genetics, University of Agriculture, Faisalabad 38040, Pakistan

**Keywords:** cotton, arabinogalactan protein, gene regulation, functional characterization

## Abstract

Arabinogalactan proteins (AGPs) constitute a diverse class of hydroxyproline-rich glycoproteins implicated in various aspects of plant growth and development. However, their functional characterization in cotton (*Gossypium* spp.) remains limited. As a globally significant economic crop, cotton serves as the primary source of natural fiber, making it essential to understand the genetic mechanisms underlying its growth and development. This study aims to perform a comprehensive genome-wide identification and characterization of the AGP gene family in *Gossypium* spp., with a particular focus on elucidating their structural features, evolutionary relationships, and functional roles. A genome-wide analysis was conducted to identify AGP genes in *Gossypium* spp., followed by classification into distinct subfamilies based on sequence characteristics. Protein motif composition, gene structure, and phylogenetic relationships were examined to infer potential functional diversification. Subcellular localization of a key candidate gene, *GhAGP50*, was determined using fluorescent protein tagging, while gene expression patterns were assessed through β-glucuronidase (GUS) reporter assays. Additionally, hormonal regulation of *GhAGP50* was investigated via treatments with methyl jasmonate (MeJA), abscisic acid (ABA), indole-3-acetic acid (IAA), and gibberellin (GA). A total of 220 AGP genes were identified in *Gossypium* spp., comprising 19 classical AGPs, 28 lysine-rich AGPs, 55 AG peptides, and 118 fasciclin-like AGPs (FLAs). Structural and functional analyses revealed significant variation in gene organization and conserved motifs across subfamilies. Functional characterization of *GhAGP50*, an ortholog of AGP18 in *Arabidopsis thaliana*, demonstrated its role in promoting epidermal hair formation in leaves and stalks. Subcellular localization studies indicated that *GhAGP50* is targeted to the nucleus and plasma membrane. GUS staining assays revealed broad expression across multiple tissues, including leaves, inflorescences, roots, and stems. Furthermore, hormonal treatment experiments showed that *GhAGP50* expression is modulated by MeJA, ABA, IAA, and GA, suggesting its involvement in hormone-mediated developmental processes. This study presents a comprehensive genome-wide analysis of the AGP gene family in cotton, providing new insights into their structural diversity and functional significance. The identification and characterization of *GhAGP50* highlight its potential role in epidermal hair formation and hormonal regulation, contributing to a deeper understanding of AGP functions in cotton development. These findings offer a valuable genetic resource for future research aimed at improving cotton growth and fiber quality through targeted genetic manipulation.

## 1. Introduction

Cotton is one of the important economic crops in tropical and sub-tropical regions of the world which provides natural fiber. The improvement of fiber quality is of great importance for the textile industry. Cotton fiber development is important for fiber quality. Different kinds of stresses influence cotton growth and development, such as drought, cold, and heat stresses [1]. Cotton fiber is a unicellular trichome differentiated from the ovule epidermis [2]. The thickness of the fiber cell is highly related to fiber quality, while excessive thickening of the cell wall leads to poor fiber quality [3,4]. Hence, further studies of the genes involved in the cell wall are crucial and are useable for improvement of these traits. It is found that several genes and hormones play roles in cell elongation and the thickness of the cell wall in cotton [5]. Fiber elongation and development are regulated by indole-3-acetic acid (IAA), abscisic acid (ABA), and methyl jasmonate (MeJA) [6]. Through controlling the cellulose synthesis induced by IAA, *GhGASA10–1* can advance fiber elongation [6], whereas fiber length is decreased due to ABA treatment and increased under ABAI treatment [7]. The up-regulation of gibberellin (GA) 20-oxidase homologous genes suggested that GA participates in the development of fibers [8]. However, there are a large number of related genes to be identified for function annotation in cotton.

AGPs are a large group of highly glycosylated proteins that are classified in the family of plant cell wall hydroxyproline-rich glycoproteins [9]. Previous research revealed that *AGP* genes are involved in the biogenesis of plant cell walls, namely in the synthesis and deposition of cellulose. AGPs are divided into various subfamilies, such as classical AGPs, AG-peptides, lysine (Lys)-rich AGPs, and FLAs by domain constitutions of core proteins [10]. AGP structure affects the synthesis and deposition of pectin and secretion of callose [11]. *AtFLA4* mutant exhibited aberrant cell growth, thinner walls, and lessening in cellulose [12]. AGPs are involved in regulation of cell wall signaling as well as structural integrity [13].

It has been determined and shown that the AGP genes are present in additional plants besides *Arabidopsis*. For instance, five *BrFLAs* are expressed in inflorescence in Chinese cabbage [14]. Some findings revealed that *AGPs* play an important role in supporting cell wall remodeling in cork oak [14]. In addition, fiber development and reactions to NaCl and phytohormones regulate expression of *GhFLA* [15], and 24 tomato *FLA* genes are responsive to ABA and MeJA [16]. PtFLAs perform various functions in poplar for wood formation which is regulated by GA signaling [17]. Previous studies suggested that *GhAGP31* could participate in the root development of cotton [18].

Hormones are important for the growth of various organs in plants [19,20], and *AGPs* can respond to various hormone signals. Previous studies suggested that *AtAGP30* functions in response to ABA and *AtFLA4* is regulated by ABA signaling [19]. However, GUS activity of *GbEXPA2* in trichomes of *Arabidopsis* is up-regulated by GA and down-regulated by ABA [20]. Functioning of *AGP* was monitored in various tissues and organs of plants [21]. Further investigation demonstrated that disruption of AGP21 in *Arabidopsis* leads to the formation of aberrant root hairs [22]. It was found that young tomato has higher concentrations of Lys-rich LeAGP1 protein than roots, young stems, leaves, and flowers [23]. Interestingly, *AtAGP18* is modestly expressed in seedlings and rosettes but highly expressed in roots, shoots, and stems [24]. *AtAGP17* and *AtAGP19* exhibit the highest expression levels in roots and flowers, with moderate abundance in stems and seedlings [25]. Likewise, over-expression of *AtAGP18* leads to observations of reduced rosette size, heightened branching, and decreased seed viability [26]. Primarily, AGPs are pivotal contributors to various aspects of plant growth and development [27]. *AtFLA11* plays roles in the formation of the secondary cell wall [28], and *FLAs* regulate the deposition of cellulose [29]. *GhAGP4* plays a significant role in the development of the cell wall, which is important for the organization and function of the cell wall [30]. Suppressing the expression of *GhAGP4* through RNA interference hinders both the initiation and elongation phases of fiber development in cotton plants [30]. *GhFLA1* affects the composition of AGP, which is useable for the initiation and elongation of fiber [30]. Thus, it is important to investigate the function of *AGPs* for improvement of fiber quality in cotton.

Quantitative trait locus (QTL) analysis is an important tool for improving cotton fiber quality, and exploiting the functioning of candidate genes placed in QTL is also meaningful [31]. The gene *Gh_A13G0395* was harbored in a QTL cluster related to fiber strength and fiber length [32]. Combining the transcript data of TM-1 [33] and quantitative Real-Time Polymerase Chain Reaction (qRT-PCR) analysis revealed that *Gh_A13G0395* were highly expressed at 20, 25, and 30 DPA [32]. In the current study, *Gh_A13G0395* is described as AGP18 in *Arabidopsis* and renamed as *GhAGP50*.

The findings from this study will provide valuable theoretical support for exploring the function of AGPs. Later, candidate gene expression was analyzed among various tissues for further experiment. The experiment demonstrated the roles of *GhAGP50* in root, stem, and leaves in *Arabidopsis*, which could reveal its biological function. Its subcellular localization, over-expression in Arabidopsis, and response to hormones can provide information to reveal its function and the mechanisms involved in growth and development in crop plants, particularly cotton. The improvement of cotton fiber quality is a major goal in cotton breeding, yet the genetic and molecular mechanisms underlying fiber development are not fully understood. AGPs, as key regulators of cell wall biogenesis and signaling, are likely to play important roles in fiber development. However, the specific functions of AGPs in cotton, particularly in fiber initiation and elongation, remain largely unexplored. This study focuses on *GhAGP50*, a candidate gene identified through QTL analysis, to elucidate its role in fiber development and its response to hormonal signals. By investigating the expression, subcellular localization, and functional characterization of *GhAGP50* in *Arabidopsis*, this study aims to provide insights into the mechanisms by which AGPs regulate fiber development and to identify potential targets for improving cotton fiber quality.

## 2. Results

### 2.1. Genome-Wide Identification and Sequence Analysis of AGP

Four subfamilies were detected, including AG peptides, FLAs, classical AGPs, and Lys-rich AGPs. Four subfamilies of proteins were discovered with the exception of the three subfamilies for *G. arboreum*. The genome of the four cotton species rendered 24, 44, 78, and 74 AGP proteins, accordingly (Tables S2 and S3). Likewise, *G. hirsutum* and *G. raimondii* had maximum molecular weights of 57.294 kDa. The results indicated the variation among features and in the cotton AGP gene family suggested a stronger necessity for these genes to operate.

### 2.2. Phylogenetic Tree and Genomic Architecture of AGP Gene Family

**Figure 1:**
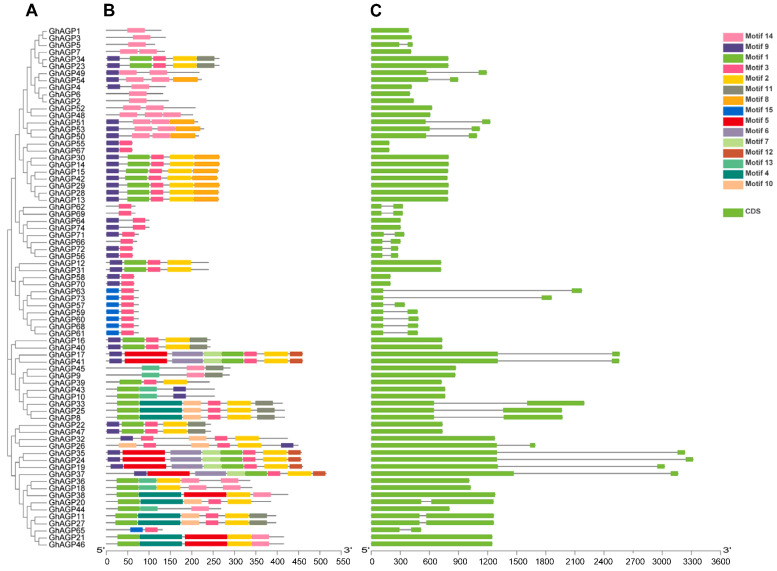
Phylogenetic and Structural Analysis of AGP Genes in *G. hirsutum*. (**A**) Phylogenetic tree illustrating the evolutionary relationships among AGP genes in *G. hirsutum*, con-structed using the Maximum Likelihood method with bootstrap values to indicate confidence levels. Different clades are highlighted in distinct colors to represent major evolutionary groups. (**B**) Conserved motif analysis displaying the distribution and arrangement of key motifs among AGP proteins. Motifs were identified using MEME, and their positions within each gene sequence are visualized, revealing similarities and differences in domain composition among AGP family members. (**C**) Gene structure analysis showing the exon-intron organization of AGP genes in *G. hirsutum*. Exons, introns, and untranslated regions (UTRs) are represented by different symbols and colors, providing insight into the structural diversity and evolutionary conservation of these genes.

There were 220 AGP proteins in total, which included 74 AGPs of *G. hirsutum* (Figure 1), 24 AGPs of *G. arboretum* (Figure S1), 78 AGPs of *G. barbadense* (Figure S2), and 44 AGPs of *G. raimondii* (Figure S3). Group I had 21 *AGPs*, whereas Group IV had 27 *AGPs*. Moreover, 19, 15, and 20 *GhAGPs* were grouped into groups I, II, and III, correspondingly. In addition, 11, 10, and 12 *GrAGPs* were categorized into groups I, II, and IV, correspondingly. Furthermore, 15 unique motifs were discovered, and protein sequences of four cotton species with significant homology retained the same patterns (Figure S4). However, there were variations in motif compositions among different subfamilies. Consequently, unique protein motifs within the subfamily are crucial for each AGP protein.

Surprisingly, genes that were closely related had stronger structural similarities despite differences in exon and intron length. Phylogeny and exon–intron structure showed a positive correlation. The results revealed the condition of exons and the gene length in the four cotton species in the *AGP* gene family.

### 2.3. Localization of AGP Genes on Chromosomes and Phylogenetic Analysis

**Figure 2:**
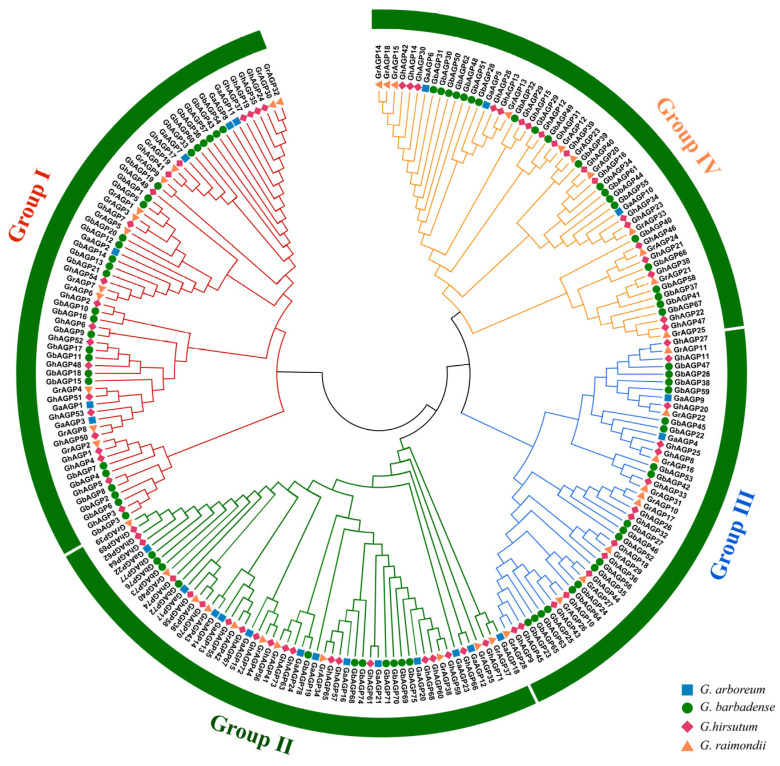
Phylogenetic analysis of AGP gene family among the four cotton species *G. hirsutum*, *G. arboreum*, *G. raimondii*, and *G. barbadense*.

The findings demonstrated that 220 cotton *AGP* genes were distributed broadly on chromosomes. There were significant differences in the number of *AGP* genes among each species. Simultaneously, *AGP* genes were found most on D07 in *G. hirsutum*, and on A07, A11, and D07 in *G. barbadense*. Among the four cotton species, the largest chromosome sizes were as follows: 135.71 Mb, 70.71 Mb, 119.88 Mb, and 103.17 Mb (Figure S5). In order to gain a deeper understanding of the evolutionary relationships among AGP proteins, an unrooted phylogenetic tree was built. Four subfamilies were identified based on evolutionary analysis among the members of cotton AGP gene family. Compared to group III, which contained the fewest AGP gene family members, group I had the most (Figure 2). The phylogenetic tree demonstrates the evolutionary relationships of the genes. Therefore, subgroups recommended for interpretation exhibit higher conservation.

### 2.4. Collinearity Analysis of AGP Genes

In *G. hirsutum*, 51,575 genes with conserved collinearity and 3049 genes with tandem repeat were discovered (Table S4). In addition, the results also revealed that 15,904 genes with conserved collinearity were in *G. arboreum* (Table S5), 5202 collinear genes were in *G. barbadense* (Table S6), and 13,754 genes with conserved collinearity were discerned in *G. raimondii* (Table S7). By the collinearity analysis of *AGP* genes between *G. arboreum* and *G. hirsutum* (Table S8), *G. arboretum* and *G. barbadense* (Table S9), *G. barbadense* and *G. raimondii* (Table S10), *G. hirsutum* and *G. raimondii* (Table S11), the quantity and percentage of collinear genes were uncovered.

**Figure 3:**
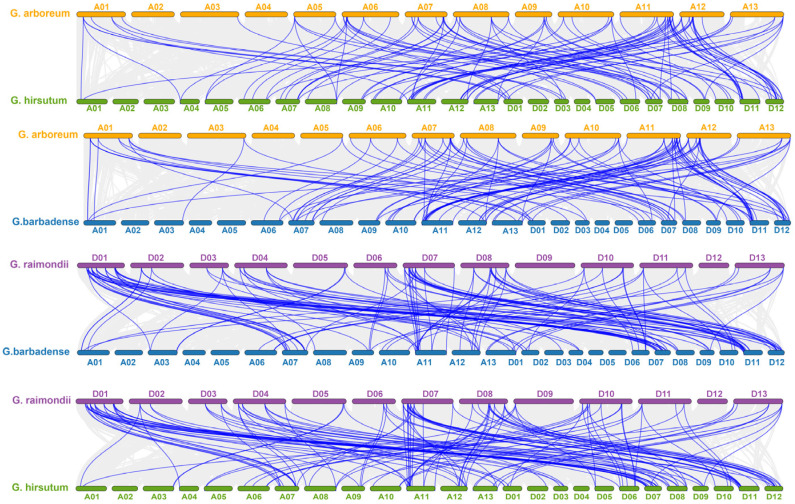
Duplication events analysis of *AGP* genes in cotton. Collinear blocks are symbolized by grey lines, while duplicated gene pairs are highlighted with blue lines.

Accordingly, it indicated that *G. hirsutum* showed 12 dispersed, 25 segmental, and five tandem gene duplications (Table S12). *G. arboreum* shared the majority of genes with collinearity on chromosomes A05, A11, and A12 (Figure 3). The result of MCScanX analysis revealed the duplication in *G. raimondii* with tetraploid cotton. Correspondingly, 118 and 115 *AGP* homologous gene duplicates were identified between diploid and tetraploid cotton (Table S13).

### 2.5. Analysis of Orthologous Gene Clusters

**Figure 4:**
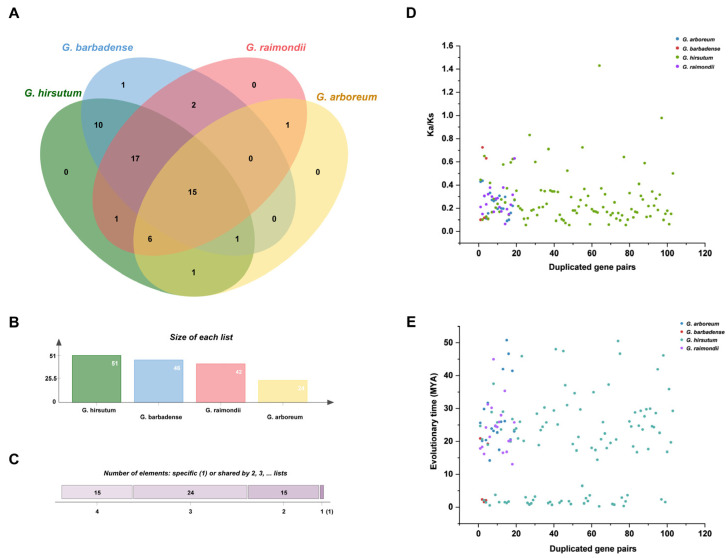
Clustering of orthologous genes and duplication events analysis of *AGP* genes in cotton. (**A**) The Venn diagram of syntenic *AGP* genes. (**B**) Number of orthologous genes. (**C**) Number of elements shared. (**D**) Synonymous and nonsynonymous ratios of *AGP* gene family. (**E**) The evolutionary time of 146 duplicated gene pairs. Ka, nonsynonymous substitution rate; Ks, synonymous substitution rate; Mya, million years ago.

Following relative assessment, the orthologous AGP gene clusters spanning the four cotton species were discovered. As a result, the cluster counts exhibited variations among the four cotton species, indicating differences in their genetic composition and diversity (Figure 4A–C). Moreover, 15 orthologous gene clusters were found. In addition, polyploidization was responsible for the emergence of unique gene clusters with particular orthologues, and orthologous gene clusters have been established. The results also revealed that in-paralogs were detected in three cotton species, the exception being *G. raimondii* (Table S14).

To explore the duplication patterns and evolutionary pressures of *AGP* paralogous gene pairs, synonymous and nonsynonymous rates were analyzed. The Ka/Ks ratio serves as a predictive measure of evolutionary history with a single value representing neutral selection. Conversely, a value less than one denotes purifying selection, while the value exceeding one signifies positive selection.

The Ka/Ks ratio in the current study was below 1 for both *AGP* orthologous and paralogous gene pairs. This suggests a scenario of purifying selection pressure accompanied by constrained functional divergence subsequent to segmental duplication. During the study, among the specific four cotton species, 146 duplicated gene pairs were identified (Figure 4D,E). Segmental duplication events involving AGP genes occurred in *G. arboreum* from approximately 14.15 to 50.78 Mya. Correspondingly, in *G. barbadense*, segmental duplication occurred from approximately 1.50–20.89 Mya. In addition, in *G. hirsutum* and *G. raimondii*, segmental duplication of *AGP* genes occurred between approximately 0.24–50.51 Mya and 13.02–44.96 Mya, respectively (Table S15).

### 2.6. cis Element Analysis of AGP Genes

The identified *cis*-regulatory elements exhibited a remarkably consistent pattern across four cotton species. ABRE, G-box, and GT1-motif were observed in comparatively greater quantities. Numerous *cis*-elements were associated with responsiveness to light and hormones (Table S16). Nine *cis*-regulatory elements were detected in *G. arboretum* (Figure S6a), *G. barbadense* (Figure S6b), and *G. raimondii* (Figure S6c) including one, two, three, two, and one elements related to ABA, GA, MeJA, auxin, and salicylic acid (SA), respectively. Besides SA, *G. hirsutum* also harbored *cis* elements associated with various other hormones (Figure S6d). Simultaneously, a variety of light-responsive *cis*-regulatory elements, i.e., GT1 motif, G-box, and AE-box, were discovered.

**Figure 5:**
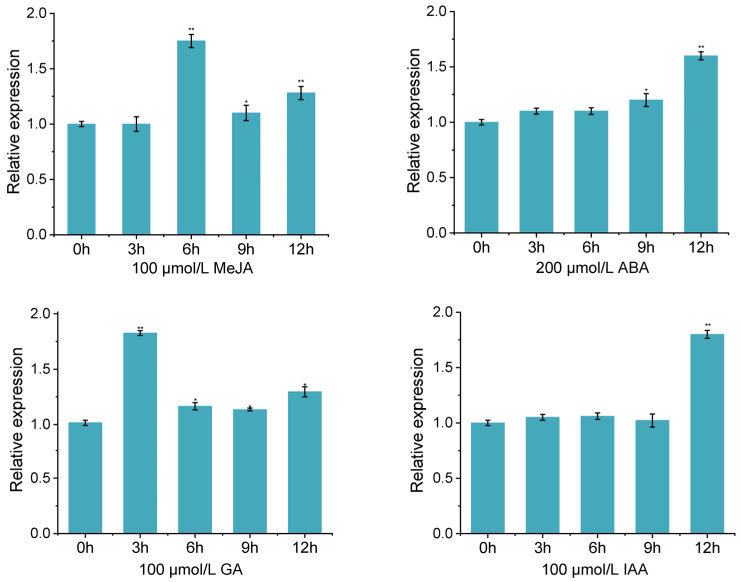
The relative expression levels of *GhAGP50* with MeJA, ABA, GA, and IAA treatment. * *p* < 0.05; ** *p* < 0.01.

To further investigate tissue-preferential and developmental regulated expression of *GhAGP50*, the 2000-bp 5′-flanking fragment upstream *GhAGP50* translation start codon (ATG) was isolated by genome walking PCR. Various putative *cis* acting regulatory elements were detected in *GhAGP50*, such as ABA responsiveness, MeJA responsiveness, auxin responsiveness, and light responsiveness (Table S16). After ABA or IAA treatment, the expression of *GhAGP50* showed the highest at 12 h (Figure 5). In addition, *GhAGP50* showed an increasing expression at 9 h and 12 h, while its expression showed highest at 6 h and 3 h after GA and MeJA treatment. These results indicated *GhAGP50* plays a role in hormone responses.

### 2.7. Subcellular Localization and Expression of Promoter::GUS Fusion of GhAGP50

**Figure 6:**
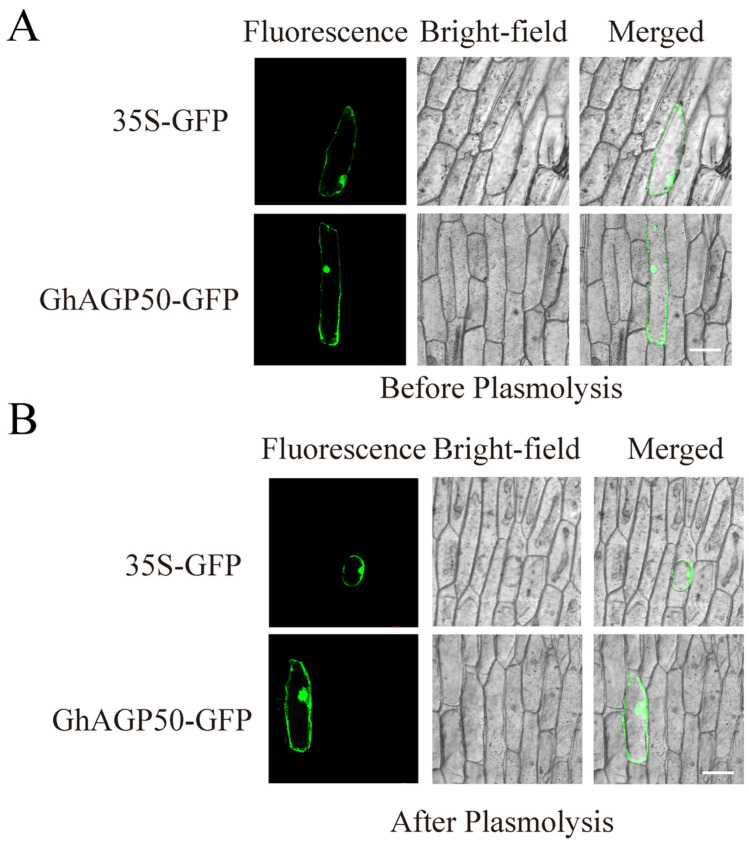
Subcellular localization of *GhAGP50*. (**A**,**B**) GhAGP50-GFP fusion protein was transiently observed in onion epidermal cells. Scale bar = 100 μm.

The gene *Gh_A13G0395* was pinpointed through a comprehensive analysis of genes localized within stable QTLs associated with fiber quality and identification of AGP genes. Based on the results of coding sequence comparison, *Gh_A13G0395* exhibits a high degree of homology to *AT4G37450*, recognized as AGP18 in *Arabidopsis*. After genome-wide identification and classification, *Gh_A13G0395* underwent renaming to *GhAGP50*. The results showed that *GhAGP50* was mainly localized in the cell nucleus and membrane. In addition, subcellular localization of *GhAGP50* was investigated in onion epidermal cells. The findings showed that fluorescence signals of GhAGP50-GFP were observed in both the cell nucleus and membrane (Figure 6 and Figure S7).

**Figure 7:**
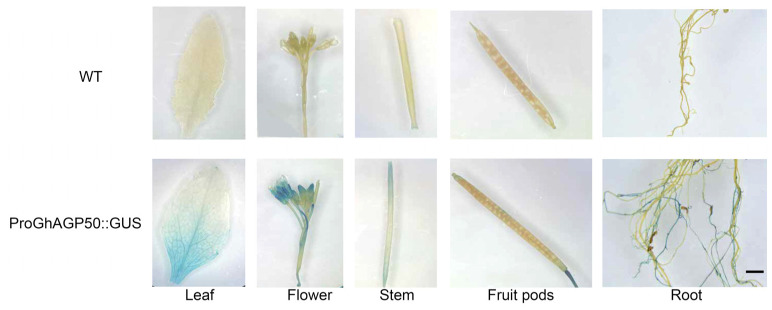
Tissue-specific expression of GhAGP50–GUS transgenic plants. Scale bar = 1 mm.

GUS activities were detected in multiple organs, including leaves, inflorescences, roots, and stems. Meanwhile, no signal was found in fruit pods and other tissues of transgenic *Arabidopsis* (Figure 7).

### 2.8. Overexpression of GhAGP50 Improved the Growth of Root Hair and Epidermal Hairs in Arabidopsis

In addition, functioning of *GhAGP50* was explored by overexpression in *Arabidopsis*. The lengths of root hairs and numbers of epidermal hairs on leaves and stalks were measured in 7-day-old wild-type, *GhAGP50*-mutant, and *GhAGP50*-overexpressing plants. In comparison to wild-type plants, overexpressed plants have longer root hairs.

**Figure 8:**
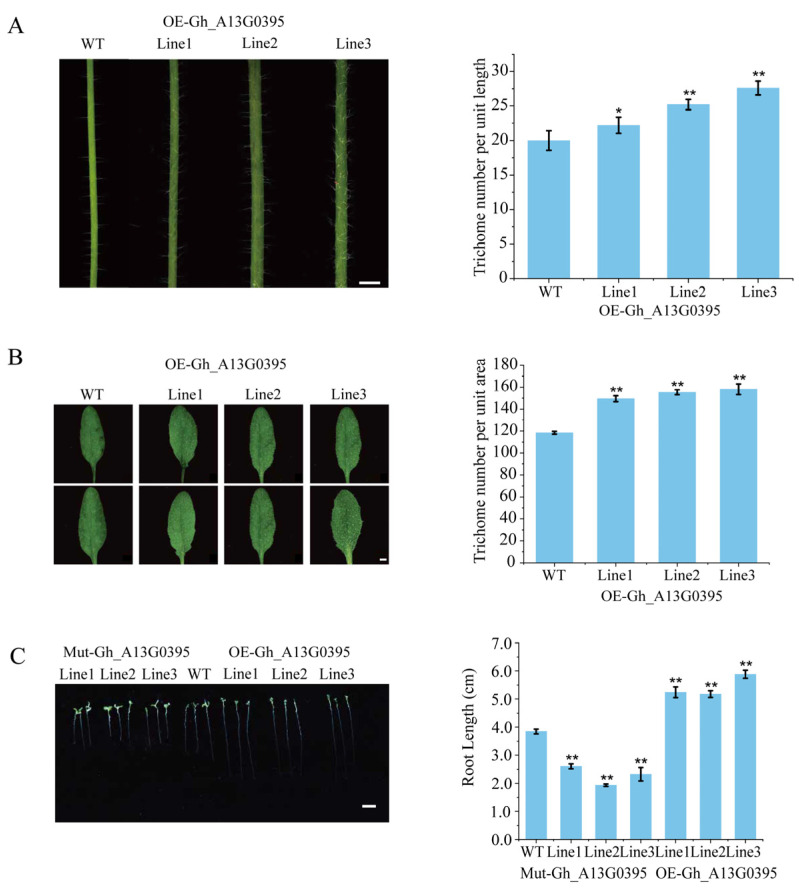
Characterization of over-expressed *GhAGP50* in *Arabidopsis*, comparing trichomes on stalks (**A**) and trichomes on leaves (**B**) between wild-type and transgenic *Arabidopsis*. Scale bars = 2 cm. (**C**) Comparing root lengths among wild-type, mutant, and transgenic *Arabidopsis*. Scale bar = 1 cm. * *p* < 0.05; ** *p* < 0.01.

Moreover, the number of epidermal hairs on leaves and stalks was increased significantly among them. In comparison to the control group, the root length of OE-*GhAGP50* lines increased by 40.40%, while Mut-*GhAGP50* lines showed a decrease of 41.30%. In addition, the numbers of trichomes on leaves and stalks increased by 30.4% and 25% in OE-*GhAGP50* lines, respectively (Figure 8).

Due to structural and genetic similarities between trichomes of *Arabidopsis* and cotton fibers, researchers have used these heterologous model species to track the activity of promoters particular to cotton fiber. *GhAGP50* overexpressed in *Arabidopsis* resulted in higher plant height than the wild type (Figure S8). Thus, *GhAGP50* can play roles in epidermal hairs of various organs in *Arabidopsis*.

## 3. Discussion

### 3.1. Evolution Analysis Revealed Purifying Selection

In the current study, 24, 78, 74, and 44 AGP genes were found in the four cotton species. The hybridization of *G. arboreum* and *G. raimondii* approximately 1–2 Mya may be the cause of the existence of most AGP genes in tetraploid cotton species [34,35]. The preservation of conserved domains throughout cotton evolution was demonstrated by comparative analysis of AGP genes. In this study, seven classical *AGPs*, 40 *FLAs*, seven Lys-rich *AGPs*, and 20 AG-peptides were identified in *G. hirsutum*. The results also revealed three classical *AGPs*, 24 *FLAs*, six Lys-rich *AGPs*, and 11 AG-peptides in *G. raimondii*, while *G. barbadense* showed nine classical *AGPs*, 46 *FLAs*, and 12 Lys-rich *AGPs* and 11 AG-peptides. During cotton fiber growth and development, many AGP carbohydrate epitopes were discovered from *G. hirsutum* L. [15,30]. The amplification of the AGP gene was primarily caused by segmental duplications. Compared to the other two cotton species, *G. arboreum* and *G. hirsutum* possessed a higher quantity of collinear blocks. Tandem and segmental duplication generally have little effect on the evolution of cotton *AGP* genes. On the other hand, the regulation of genomic diversity was dependent on purifying selection that was applied to pairs of duplicated AGP genes in wild populations [36]. Moreover, purifying selection has been demonstrated in many plants, namely rice [37], soybean [38], wheat [39], cucumber [40], cocoa [41], and cotton [42]. Despite the fact that *G. hirsutum* (AD1) and *G. barbadense* (AD2) were the initial species to undergo transoceanic hybridization, this evolutionary process commenced about 1–2 million years ago [43].

### 3.2. The Evolutionary Events of AGPs Influence Functional Diversification

The AGP gene family has been expanded through gene duplication and differentiation events with differentiating functionally and adapting to the different physiological and ecological needs of plants. This gene family expansion and functional differentiation is an important mechanism for adaption to various environmental stresses [44]. AGP family genes contain functional domains, such as the fasciclin domain and the arabinogalactan domain, with different structures and functions. The diversity of these functional domains allows AGP proteins to perform multiple functions in processes such as cell wall construction, cell signaling, and cell–cell interactions [45]. The functional variety of AGP genes is further increased by variations in their expression patterns throughout different developmental stages and tissues. Plant growth and environmental adaptability are significantly influenced by certain AGP genes, which are strongly expressed during particular developmental stages or in response to particular environmental stimuli [44].

### 3.3. Regulation of AGPs by Various Hormones During Plant Growth

AGPs are not only significant cell wall components but also important nutritional or signaling molecules [46]. Although the precise processes are still unclear, *AGPs* have also been associated with signal transduction and recognition [47]. ABA is an important hormone, while several *AGPs* are responsive to ABA and salt stresses in rice [48]. Furthermore, *AGPs* are important in α-amylase synthesis that GA induced in wheat cells [48]. In *Brassica rapa*, ABA, GA, and MeJA treatments resulted in up-regulation of 17 *BrAGPs* and down-regulation of six *BrAGPs* [49]. *AmAGP* is expressed high with IAA treatment in *Amorphophallus mueller* [50]. IAA and MeJA promote fiber elongation, while ABA inhibits fiber elongation. Likewise, GA could promote cotton fiber elongation and ovule expansion. In this study, expression of *GhAGP50* was regulated by various concentrations of GA, IAA, ABA, and MeJA. The interaction of GA with auxins orchestrates the differentiation and growth of epidermal hairs, ensuring proper development and functionality [21]. ABA inhibits root hair formation in epidermal cells [51]. MeJA plays a significant role in the initiation and elongation of root hairs [11]. *GhAGP50* might be influenced by these hormones to regulate epidermal hair formation and development. By participating in cell expansion, differentiation, and interaction with other cellular components, AGPs play a crucial role in regulating the formation of root and shoot epidermal hairs. AGPs that are recognized by specific antibodies are essential for these developmental processes [52].

### 3.4. AGPs Play Roles in the Growth and Development of Plants

AGP is one of the most intricate kinds of macromolecules [53]. In addition, AGPs are complex hydroxyproline-rich glycoproteins, which affect the structure and composition of the cell wall [10]. *AGPs* are involved in diverse processes of plant development, including cellular differentiation, signaling, and interaction between microbes and plants [54]. In addition, *AtAGP19* promoter-controlled GUS activity was identified in the veins of leaves, roots, stems, styles, and siliques [55]. In this study, *GhAGP50* was found to be expressed in leaves, inflorescences, roots, and stems, suggesting its potential function in controlling growth and development of plants. AGPs play crucial roles in root growth and regeneration, root hair formation, and seedling growth [56]. In this study, the numbers of epidermal hairs on the stems and leaves of transgenic *Arabidopsis* were greater than those of wild-type *Arabidopsis*, demonstrating that *GhAGP50* promotes the formation of epidermal hairs. Thus, it is important for further exploration to investigate the function of *AGP*.

### 3.5. AGPs Regulate Cotton Fiber Development

In *Arabidopsis*, traditional AGP18 actively regulates survival and megaspore selection [57]. *AGPs* are located on plasma membranes and AGPs play important roles in various biological processes [14]. Fiber elongation to transitional cell-wall remodeling is important for the development of cotton fiber [58]. AGPs are highly correlated with cell wall components, which play roles in the fiber initial differentiation and elongation developmental stages [30]. Previous researchers reported that *AGPs* are involved in the elongation of fiber, which plays a role in improving cotton fiber quality [59]. The gene *GhGalT1* controls glycosylation of AGP to regulate cotton fiber elongation [60]. The AG glycan on AGP plays a role in controlling fiber development, which shows the importance of discovering genes related to specific glycans to improve fiber quality [60]. In this study, several identified *AGP* genes were significant for further study for fiber development in cotton.

## 4. Materials and Methods

### 4.1. Identification and Characterization of AGP Genes

The genomic sequences of *G. arboreum* (CRI), *G. raimondii* (JGI), *G. barbadense* (ZJU) and *G. hirsutum* (NAU) were from the CottonFGD database and CottonGen database [61]. HMMER software v3.4 with an E value threshold of 1 × 10^−10^ utilized Hidden Markov Models (HMMs) for the detection of potential AGP sequences [62]. The HMM files (PF02469 and PF06376) were used to identify *FLAs* and AG-peptides. Protein domains (IPR044959 and IPR044981) were used to detect classical and Lys-rich *AGPs*. In addition, the Pfam (https://pfam.xfam.org/) and InterPro (https://www.ebi.ac.uk/interpro/) databases were utilized to verify the accuracy of the identified candidate protein sequences. The biophysical properties of AGP proteins underwent examination with an online tool, i.e., Expasy [63]. Bologna Unified Subcellular Component Annotator (BUSCA) was used to predict subcellular localization of proteins [64].

### 4.2. Phylogenetic Investigation and Gene Structure of AGP Genes

The *AGP* sequences from four cotton species were under alignment using Clustal Omega (version 1.2.4) with standard parameters [65]. The generated multiple sequence alignments (MSAs) were constructed in the MEGA 7 software and then visualized for analysis [66]. The identification of conserved motifs was carried out using MEME [67], while the MAST algorithm was used to examine the protein database for 15 motifs [68]. Gene structure was determined by evaluating both coding sequences and genomic sequences. TBtools was employed to visualize the gene structure of AGPs [69]. To construct a phylogenetic tree, AGP proteins were analyzed in MEGA 7 [66].

### 4.3. Chromosomal Locations and Gene Collinearity Analysis of AGP Genes

Chromosome locations of *AGP* genes were drawn with TBtools software (Version 2.027) [69]. *AGP* genes were given systematic nomenclature depending on the location of their chromosomes. The assessment of gene duplication occurrences involved the utilization of the MCScanX toolkit, which performed multiple collinearity scans for analysis [70]. The visualization of duplicated regions involved illustrating the syntenic relationships among AGP genes using Circos. Tandem duplicates are gene pairs that are duplicates of each other, originating from same genome, and located on same chromosome [71]. TBtools facilitated the computation of selection pressure by evaluating the ratio of nonsynonymous (Ka) to synonymous (Ks) substitutions. The duplication time (T) in evolutionary analysis was computed using the formula T = Ks/2λ × 10^−6^ (Mya), with a λ value of 1.5 × 10^−8^ [72].

### 4.4. Identifying of cis Regulatory Element

In the cotton AGP promoter region, the 1 kb promoter was to pinpoint the important *cis*-regulatory components. The sequence was supplied form the CottonFGD database. PlantCARE (accessed on 1 April 2025) was used to determine *cis*-regulatory elements [73].

### 4.5. Subcellular Localization

The XbaI and SmaI restriction sites on the pCAMBIA2300 vector were analyzed to build the translation fusion structure. As part of the experimental procedure, the recombinant plasmid experienced transformation into *Agrobacterium tumefaciens* GV3101. *Nicotiana benthamiana* (tobacco) leaves were concurrently transformed with the pCAMBIA2300 vector to serve as the control group under identical conditions. The observation of the transformed gene expression was conducted using a CCD optical microscope (Germany’s Leica Microsystems, Wetzlar, Germany).

To investigate subcellular localization of *GhAGP50*, the PDS-1000/He system (Bio-Rad, Hercules, CA, USA) facilitated the transformation of onion epidermal cells with both the empty vector and recombinant plasmids containing GFP fusions. Following a 24–36 h incubation period on MS media, the fluorescence of GFP was detected using confocal microscopy. The 30% sucrose solution was applied to the cells to perform the plasmolysis experiment.

### 4.6. GhAGP50 Promoter Expression in Transgenic Arabidopsis Plants

A GUS reporter cassette was utilized to assess the promoter activity. It contained the *GhAGP50* promoter inserted into the SalI/BamHI sites of the pBI121 vector. The GhAGP50::GUS construct was introduced into *A. tumefaciens* GV3101 using normal transformation techniques. The selection of positive plants was carried out using MS plates supplemented with kanamycin at the concentration of 50 g/mL. Using the GUS Stain Kit (Solarbio, Beijing, China, G3061), histochemical tests were performed to determine the GUS activity in transgenic *Arabidopsis*. A Leica stereomicroscope (Leica MZ16f) was utilized for examining and photographing the stained tissue.

### 4.7. Transformation in Arabidopsis

Wild-type *Arabidopsis* type Columbia Zero and *Arabidopsis* mutant (SALK_117268) were acquired from Nottingham *Arabidopsis* Stock Centre (NASC). *Arabidopsis* was transformed using the floral dip approach after the pBI121 vector was introduced into *A. tumefaciens* GV3101. The *GhAGP50* ORF was integrated into the pBI121 vector under the direction of cauliflower mosaic virus (CaMV) 35S promoter. The experiments utilized the T3 generation for further investigations. To determine the positivity of the offspring seedlings, PCR amplification was used to detect the target gene. *Arabidopsis* seeds of the wild type, genetically modified to include pBI121–GhAGP50, underwent sterilization before being placed onto MS medium for growth. The phenotypes of the planted *Arabidopsis* were evaluated and analyzed when reaching maturity.

### 4.8. Exogenous Hormone Treatment

Upon reaching the three-leaf stage, a portion of the cotton plants were designated as the control group, while the remaining plants were treated with 100 μmol/L MeJA, 200 μmol/L ABA, 100 μmol/L GA, and 100 μmol/L IAA. Each treatment was replicated three times biologically. Leaf tissue samples were obtained at 3 h intervals following treatment, spanning 3 h, 6 h, 9 h, and 12 h. RNA extraction was then performed on these samples to assess gene expression levels. The cotton material treated was 0–153, with high-quality fiber.

### 4.9. RNA Extraction and qRT-PCR Analysis

The material used for RNA extraction was 0–153. Leaf tissue RNA extraction was conducted using an RNA extraction kit, FastPure Universal Plant Total RNA Isolation Kit (Vazyme Biotech Co., Ltd., Nanjing, China). To validate the expression pattern, qRT-PCR was conducted. HiScript^®^ II Q RT SuperMix for qPCR (+gDNA wiper) kit (Vazyme, R223-01) was used to reverse transcribe 1 μg RNA of each sample into cDNA. Specific primers were designed using Primer Premier 5 and the housekeeping gene *GhHis3* was a reference gene in this experiment (Table S1) [74]. The relative expression levels of candidate genes were computed by the 2^−ΔΔCT^ method and the bar graph was plotted using OriginLab [75]. The statistical significance was analyzed by using two-tailed tests.

## 5. Conclusions

In order to highlight the potential functional diversity, whole-genome identification and evolutionary analysis were performed. In addition, phylogenetic relationships and expression patterns were analyzed for the cotton *AGP* gene family. These results could contribute to understanding the functional characteristics of cotton *AGPs*. *GhAGP50* was harbored in a fiber length and fiber strength QTL, and identified as AGP18, which is an important protein composing the plant cell wall. In this study, overexpression of *GhAGP50* promoted epidermal hair of leaves and stems, which means it contributes to regulating cotton fiber development. This study contributes to the understanding of the roles of *AGPs* in improving cotton fiber quality and the processes of plant growth and development.

## Data Availability

All data generated or analyzed during this study are included in this manuscript and its Supplementary Information Files.

## Supplementary Materials

The supporting information can be downloaded at: https://www.mdpi.com/article/10.3390/ijms26094159/s1.
