# Supplementary material for: Genome-Wide Identification and Classification of Arabinogalactan Proteins Gene Family in Gossypium Species and GhAGP50 Increases Numbers of Epidermal Hairs in Arabidopsis
Source: Int J Mol Sci. 2025 Apr 27;26(9):4159. doi: 10.3390/ijms26094159 (PMC12071561; doi:10.3390/ijms26094159)
Supplement: Supplementary file 1 [file ijms-26-04159-s001.zip › Supplymentaty Figure/Figure S4.pdf]

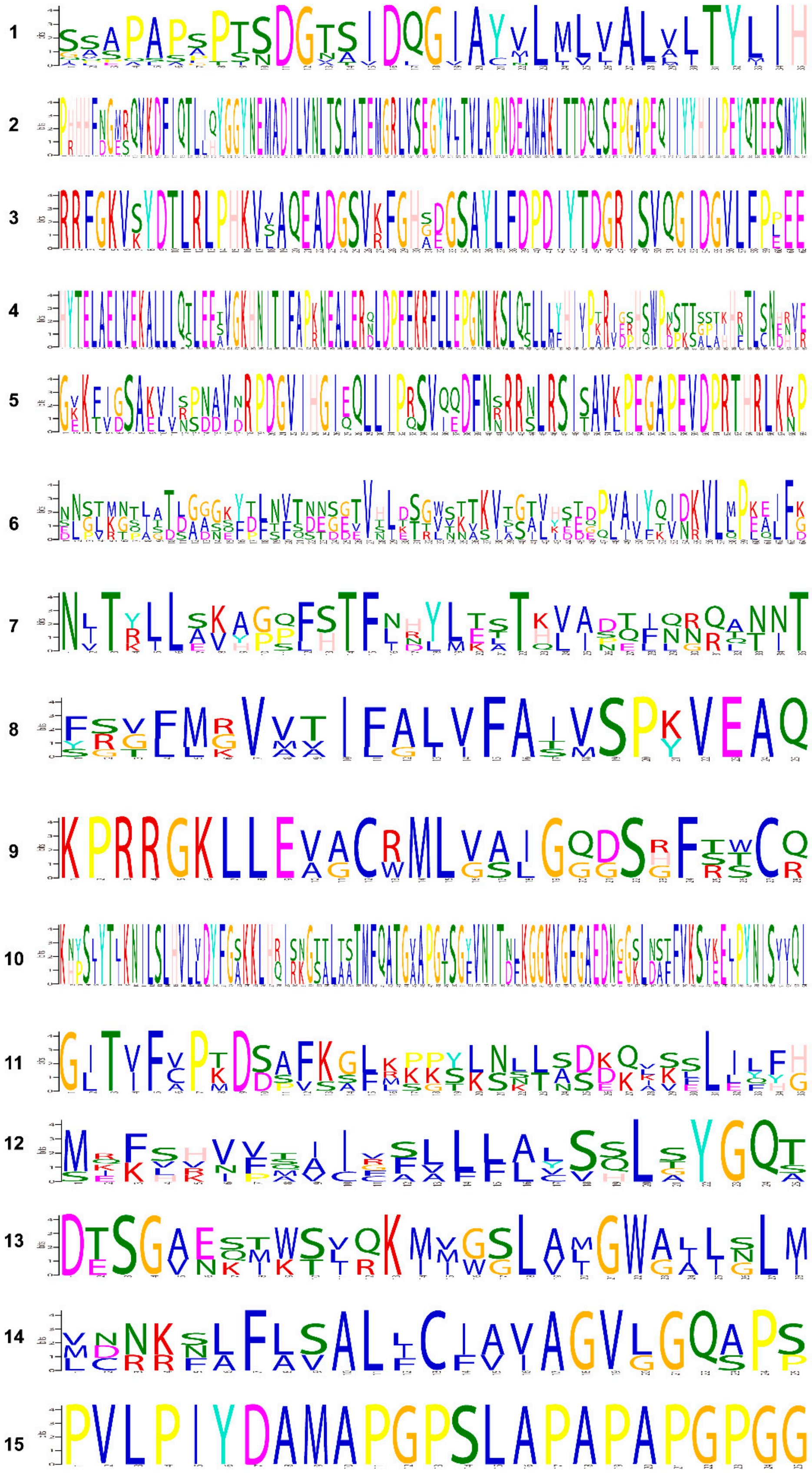

Supplementary Fig. 4a. Sequence logos of conserved motifs in *G. arboreum* AGP proteins.

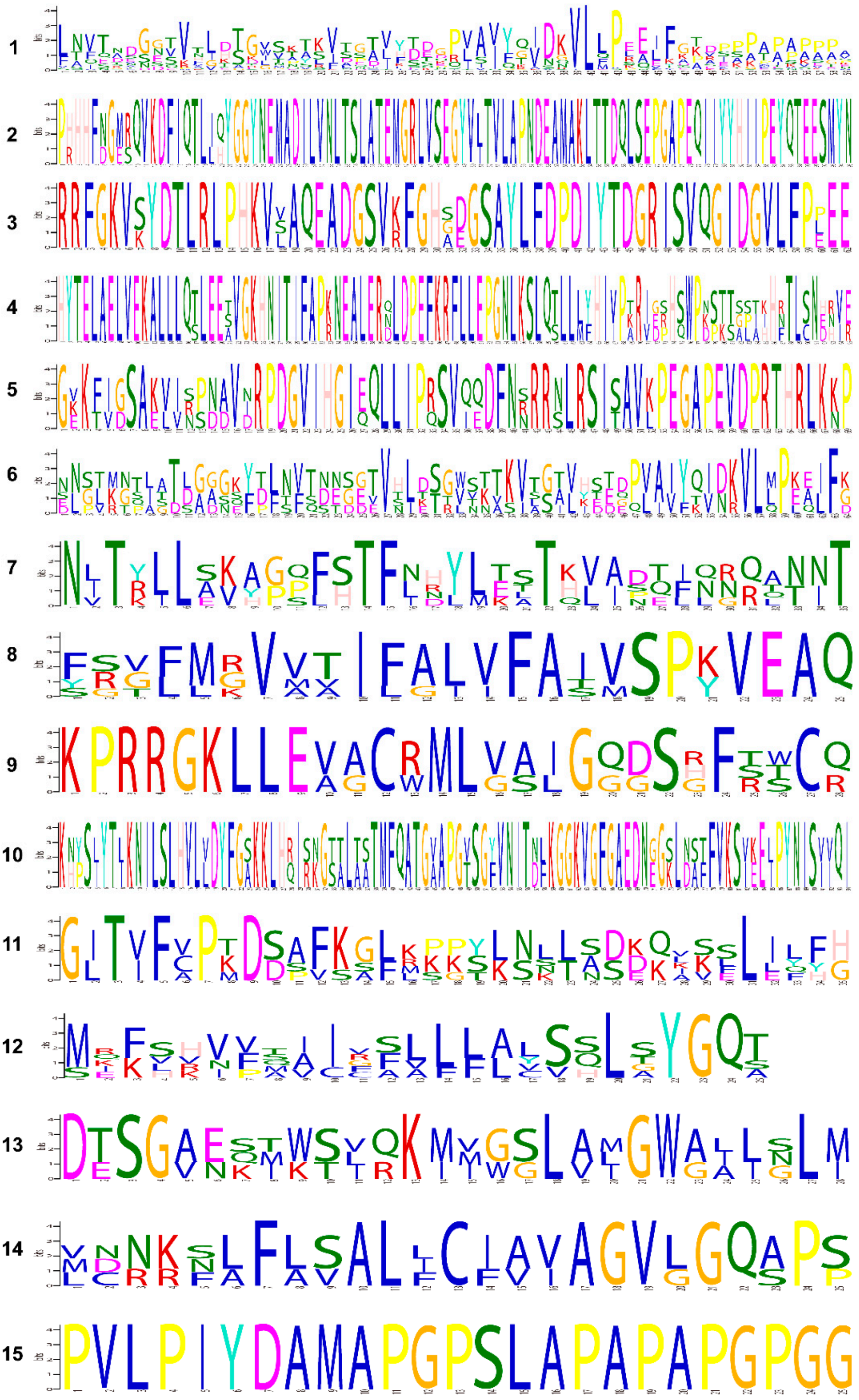

Supplementary Fig. 4b. Sequence logos of conserved motifs in *G. barbadense* AGP proteins.

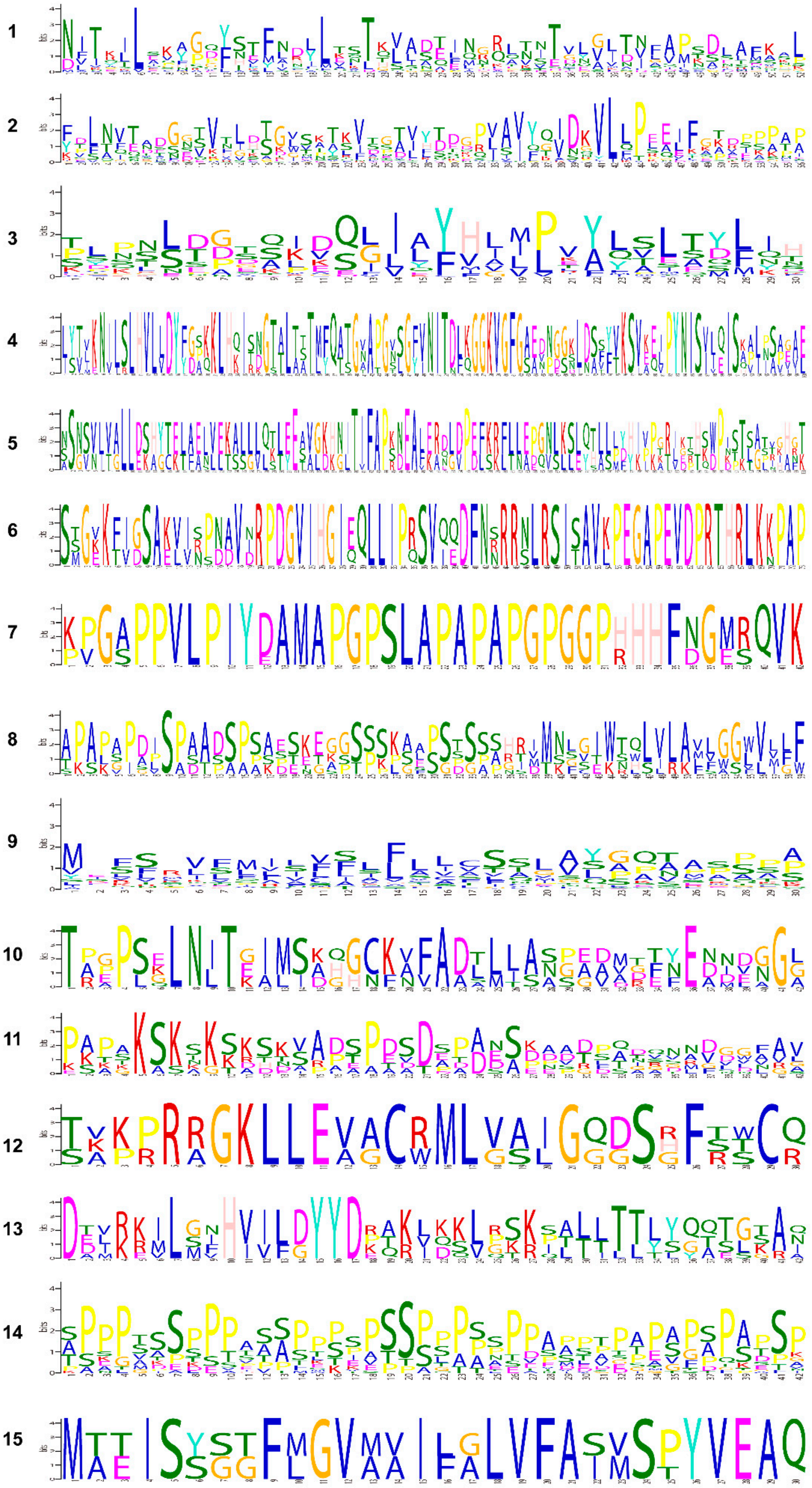

Supplementary Fig. 4c. Sequence logos of conserved motifs in *G. hirsutum* AGP proteins.

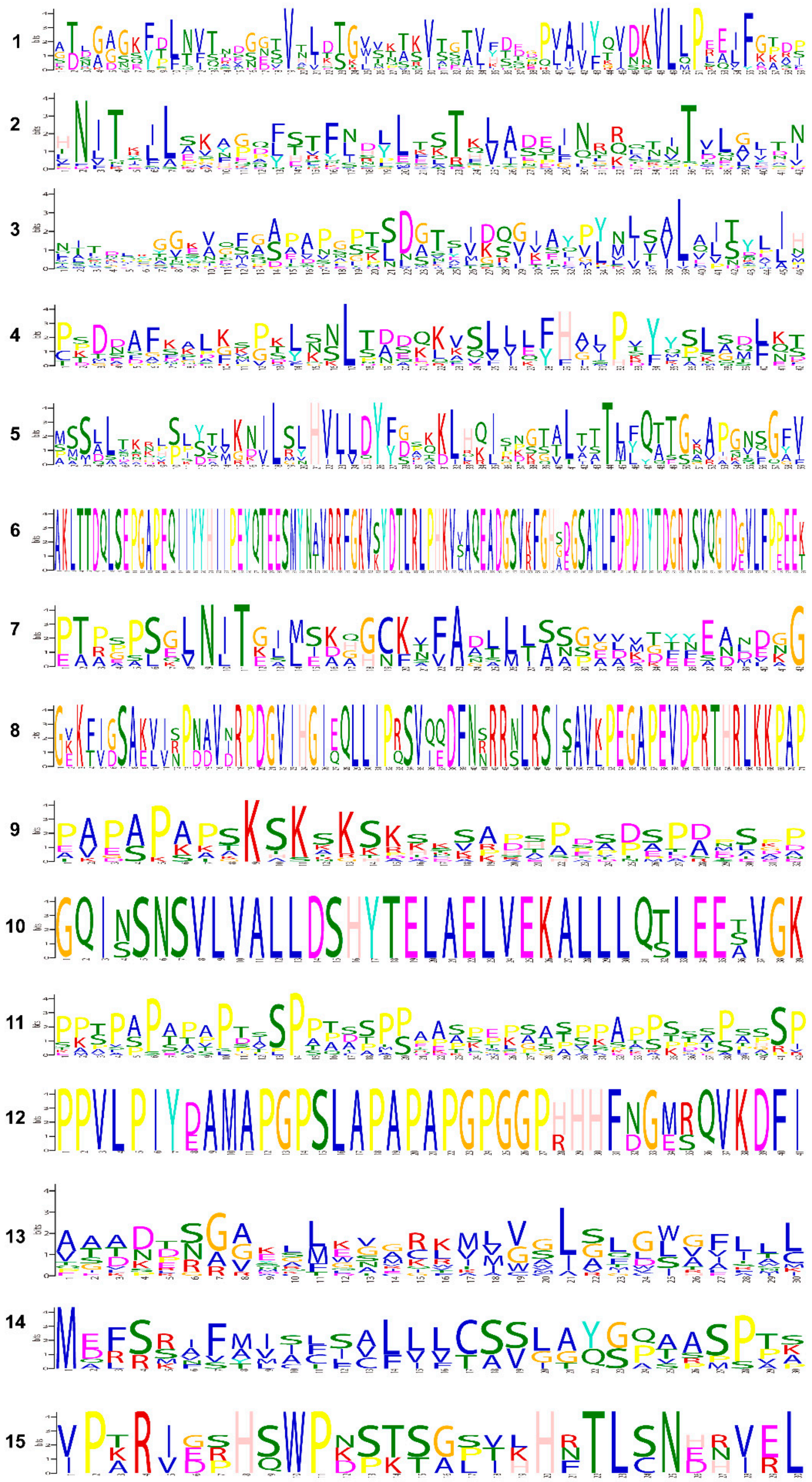

Supplementary Fig. 4d. Sequence logos of conserved motifs in *G. raimondii* AGP proteins.p
