# Supplementary material for: Genome-Wide Identification and Classification of Arabinogalactan Proteins Gene Family in Gossypium Species and GhAGP50 Increases Numbers of Epidermal Hairs in Arabidopsis
Source: Int J Mol Sci. 2025 Apr 27;26(9):4159. doi: 10.3390/ijms26094159 (PMC12071561; doi:10.3390/ijms26094159)
Supplement: Supplementary file 1 [file ijms-26-04159-s001.zip › Supplymentaty Figure/Figure S6d.pdf]

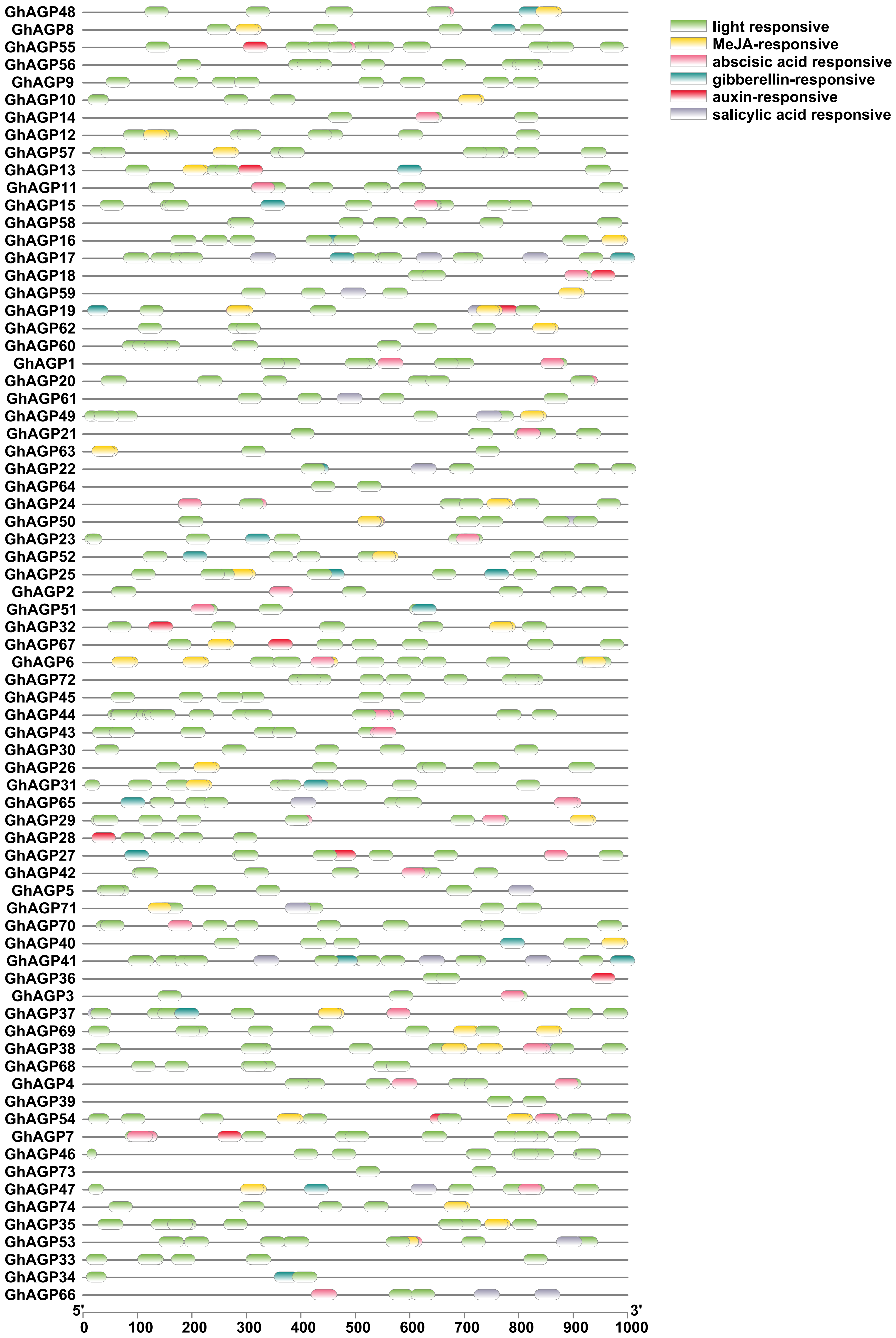
GhAGP48
GhAGP8
GhAGP55
GhAGP56
GhAGP9
GhAGP10
GhAGP14
GhAGP12
GhAGP57
GhAGP13
GhAGP11
GhAGP15
GhAGP58
GhAGP16
GhAGP17
GhAGP18
GhAGP59
GhAGP19
GhAGP62
GhAGP60
GhAGP1
GhAGP20
GhAGP61
GhAGP49
GhAGP21
GhAGP63
GhAGP22
GhAGP64
GhAGP24
GhAGP50
GhAGP23
GhAGP52
GhAGP25
GhAGP2
GhAGP51
GhAGP32
GhAGP67
GhAGP6
GhAGP72
GhAGP45
GhAGP44
GhAGP43
GhAGP30
GhAGP26
GhAGP31
GhAGP65
GhAGP29
GhAGP28
GhAGP27
GhAGP42
GhAGP5
GhAGP71
GhAGP70
GhAGP40
GhAGP41
GhAGP36
GhAGP3
GhAGP37
GhAGP69
GhAGP38
GhAGP68
GhAGP4
GhAGP39
GhAGP54
GhAGP7
GhAGP46
GhAGP73
GhAGP47
GhAGP74
GhAGP35
GhAGP53
GhAGP33
GhAGP34
GhAGP66
5'
3'
0
100
200
300
400
500
600
700
800
900
1000
light responsive
MeJA-responsive
abscisic acid responsive
gibberellin-responsive
auxin-responsive
salicylic acid responsive
