# Supplementary figures and images for: Genome-Wide Identification and Classification of Arabinogalactan Proteins Gene Family in Gossypium Species and GhAGP50 Increases Numbers of Epidermal Hairs in Arabidopsis
Source: Int J Mol Sci. 2025 Apr 27;26(9):4159. doi: 10.3390/ijms26094159 (PMC12071561; doi:10.3390/ijms26094159)

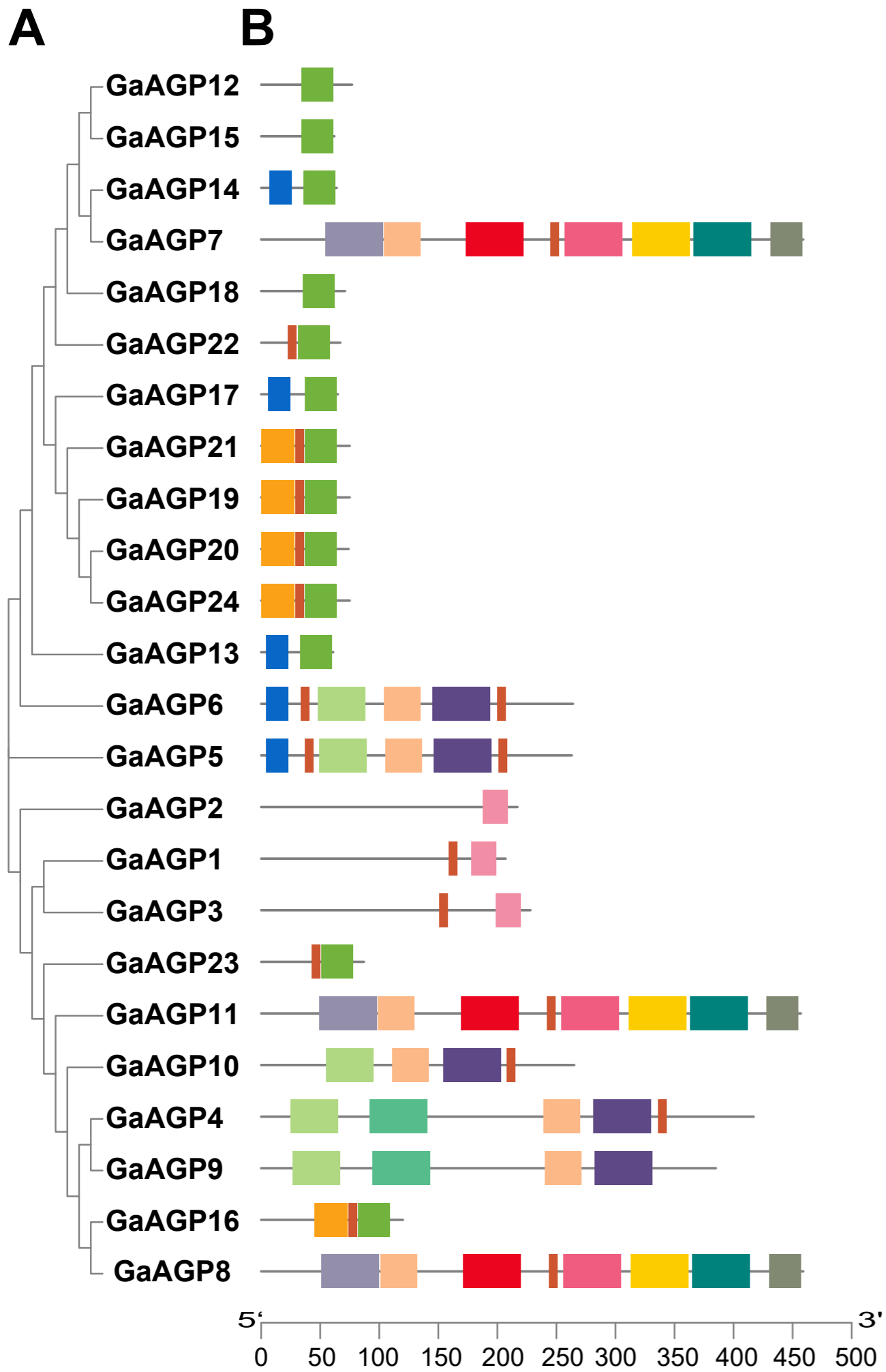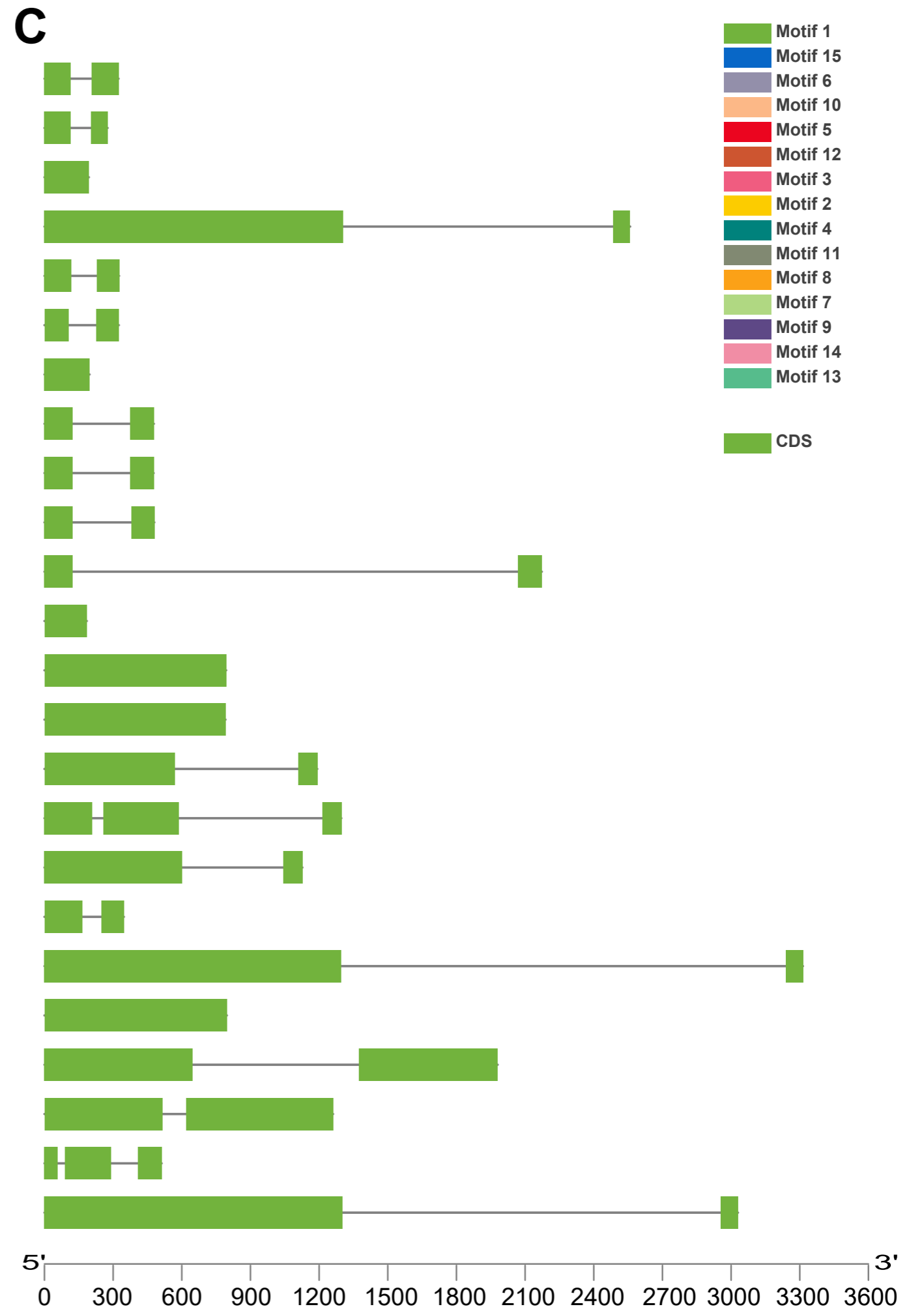

Supplement: Supplementary file 1 [file ijms-26-04159-s001.zip › Supplymentaty Figure/Figure S1.pdf]

A

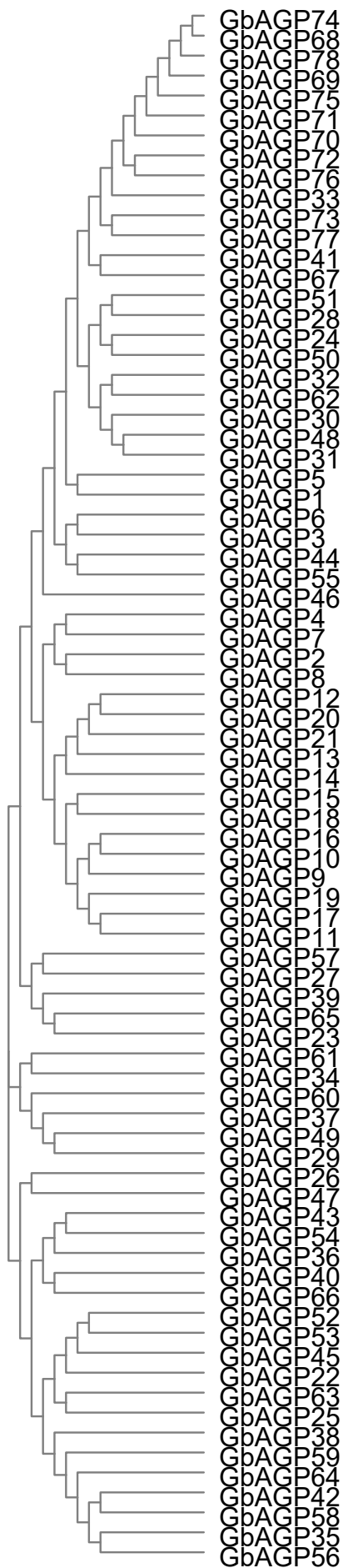

B

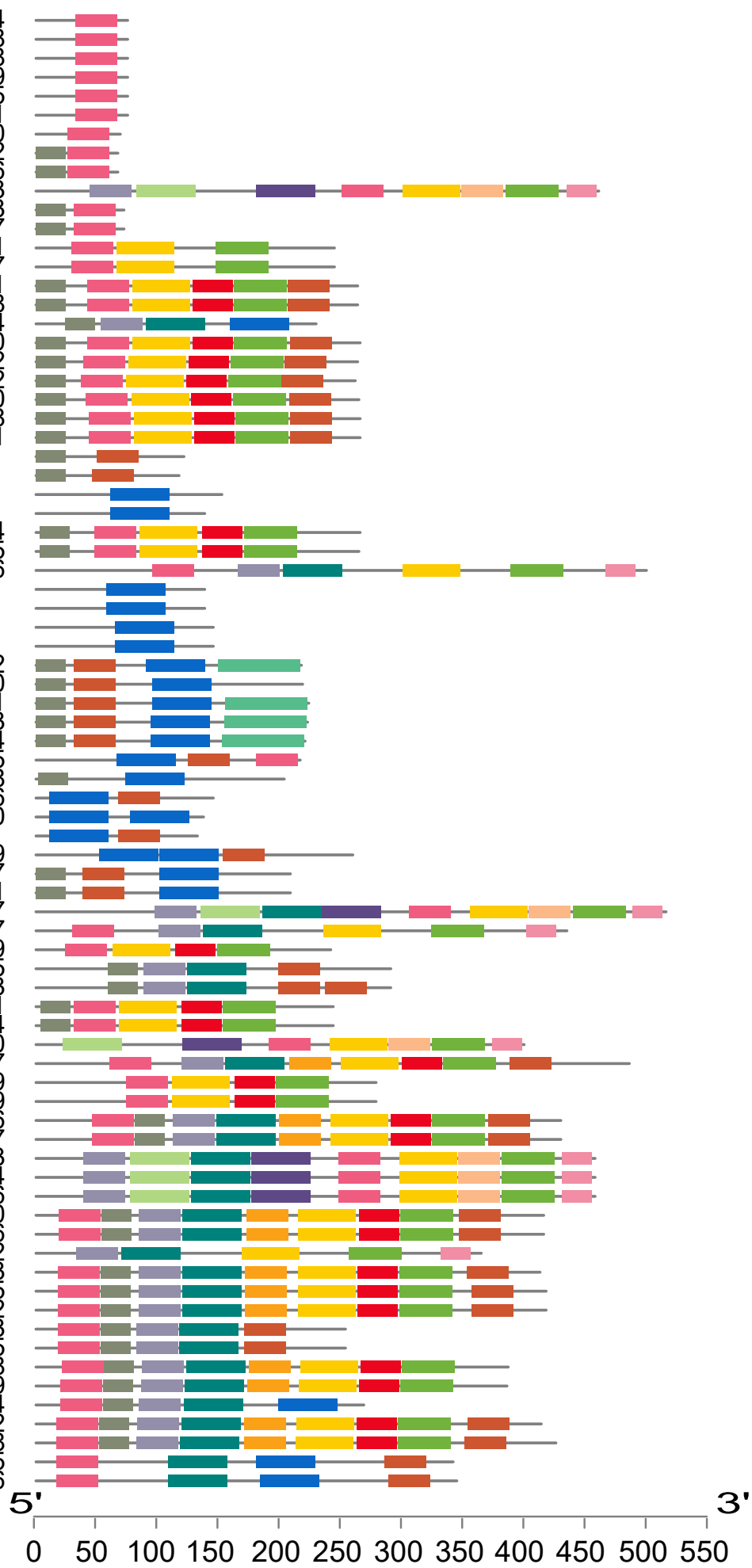

C

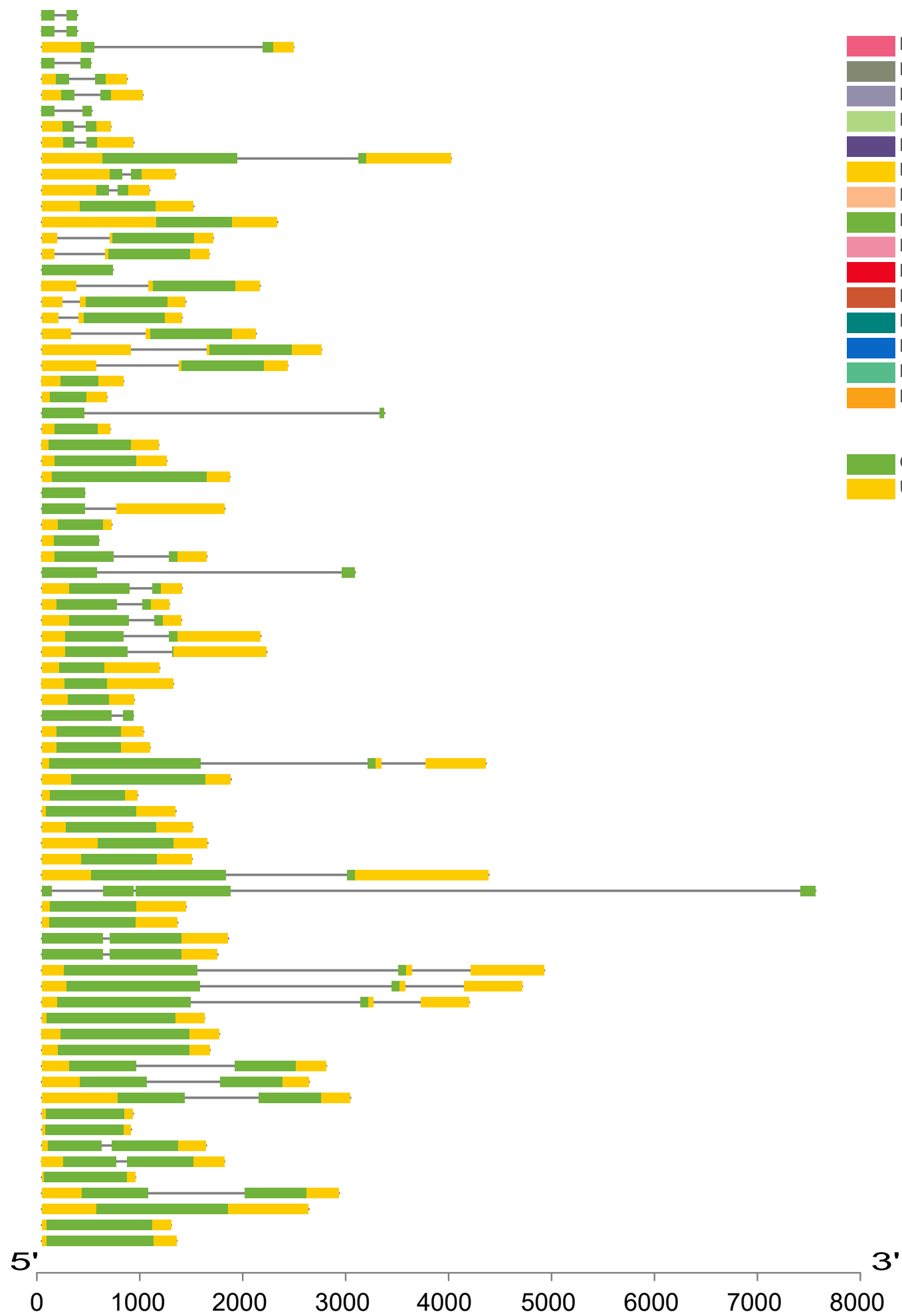

Supplement: Supplementary file 1 [file ijms-26-04159-s001.zip › Supplymentaty Figure/Figure S2.pdf]

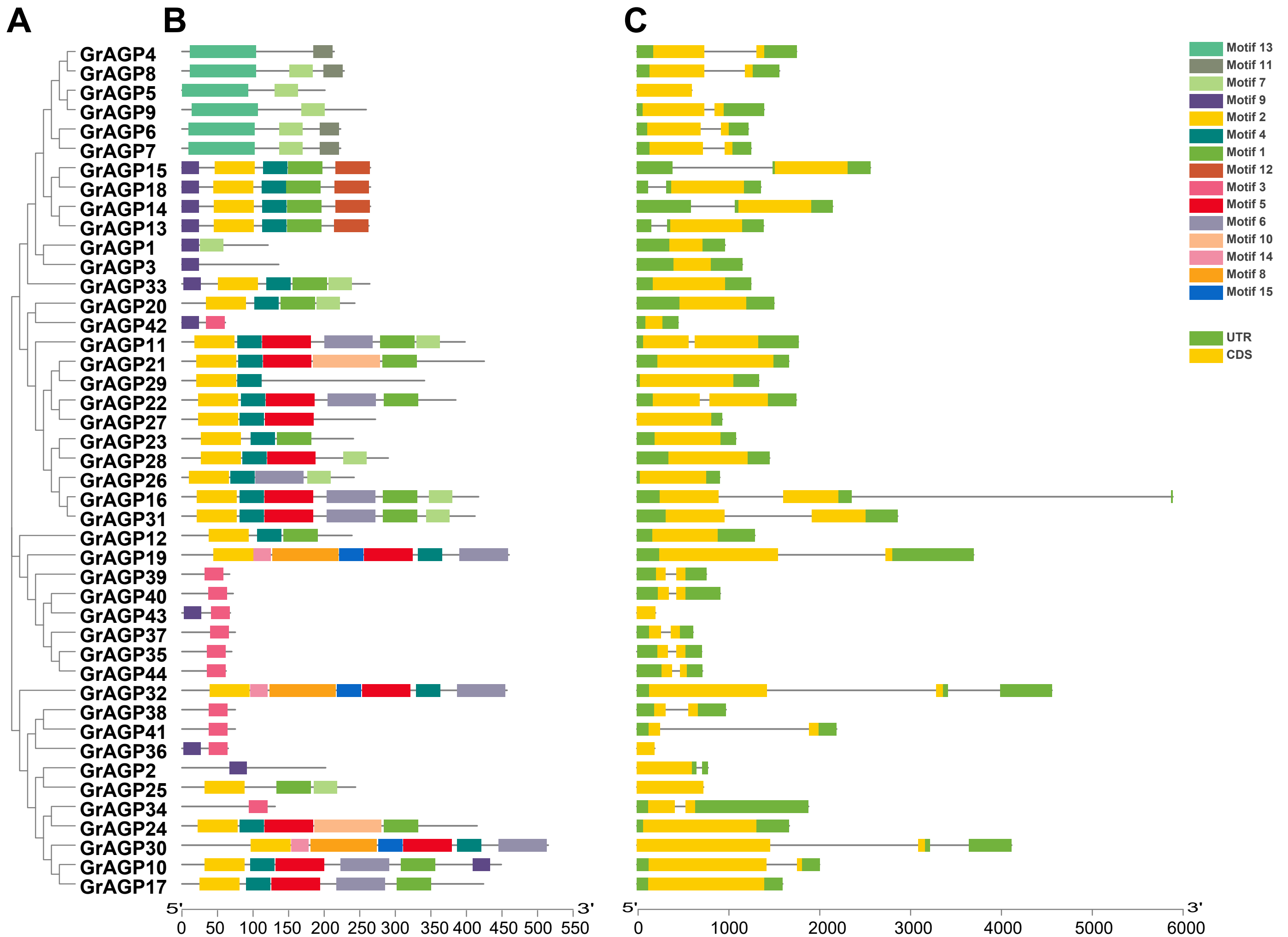

Supplement: Supplementary file 1 [file ijms-26-04159-s001.zip › Supplymentaty Figure/Figure S3.pdf]

A

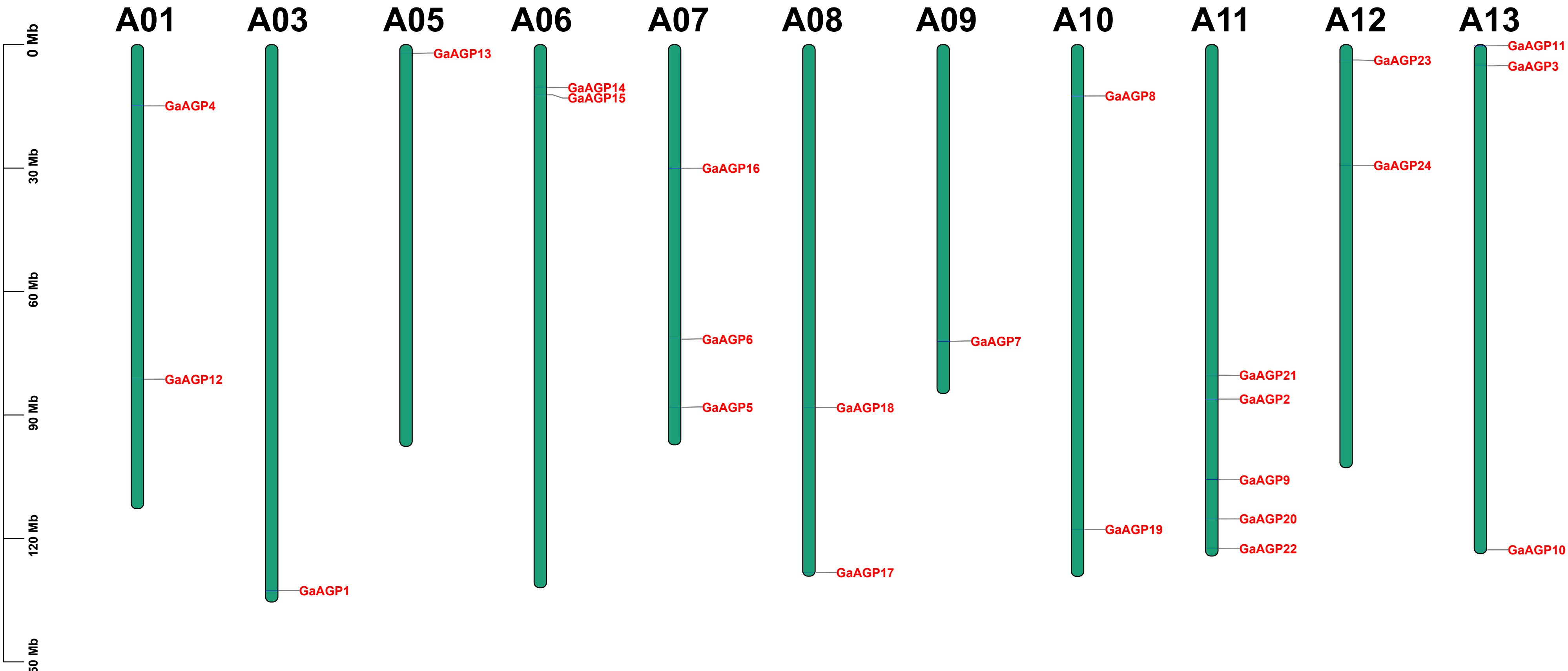

B

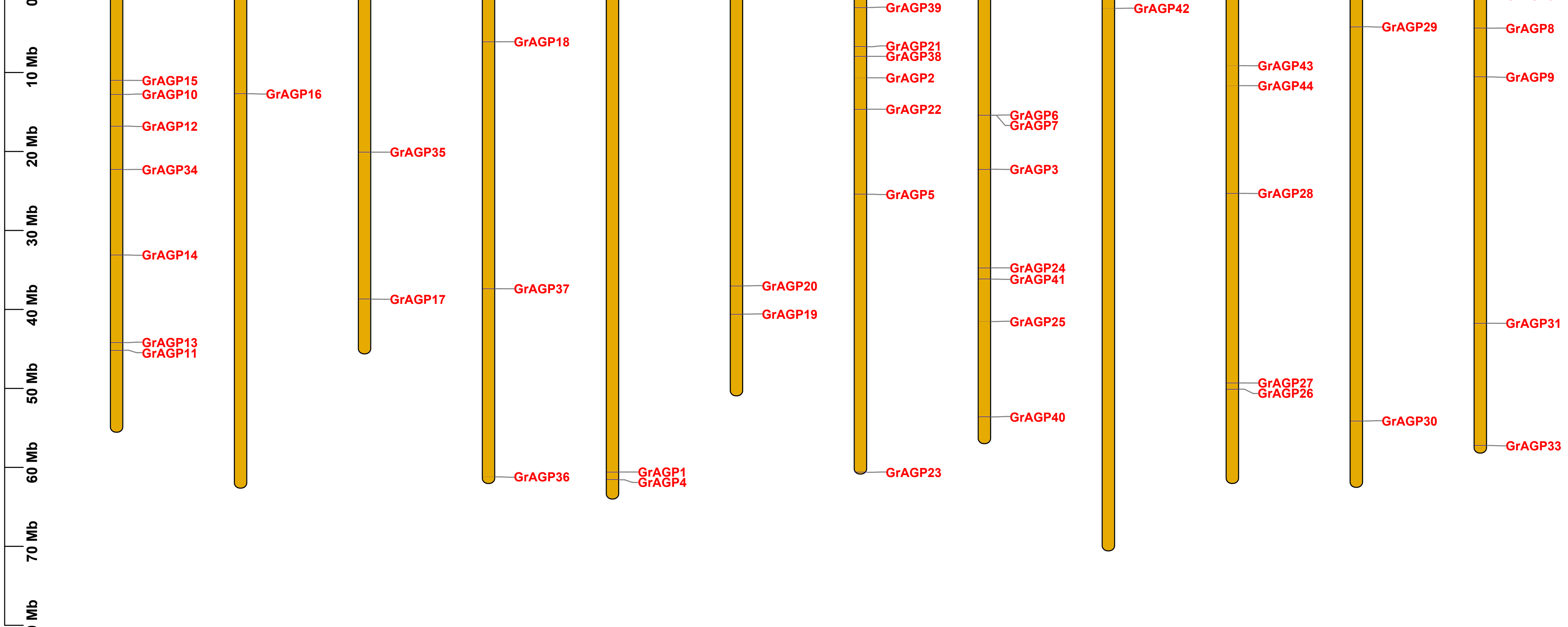

C

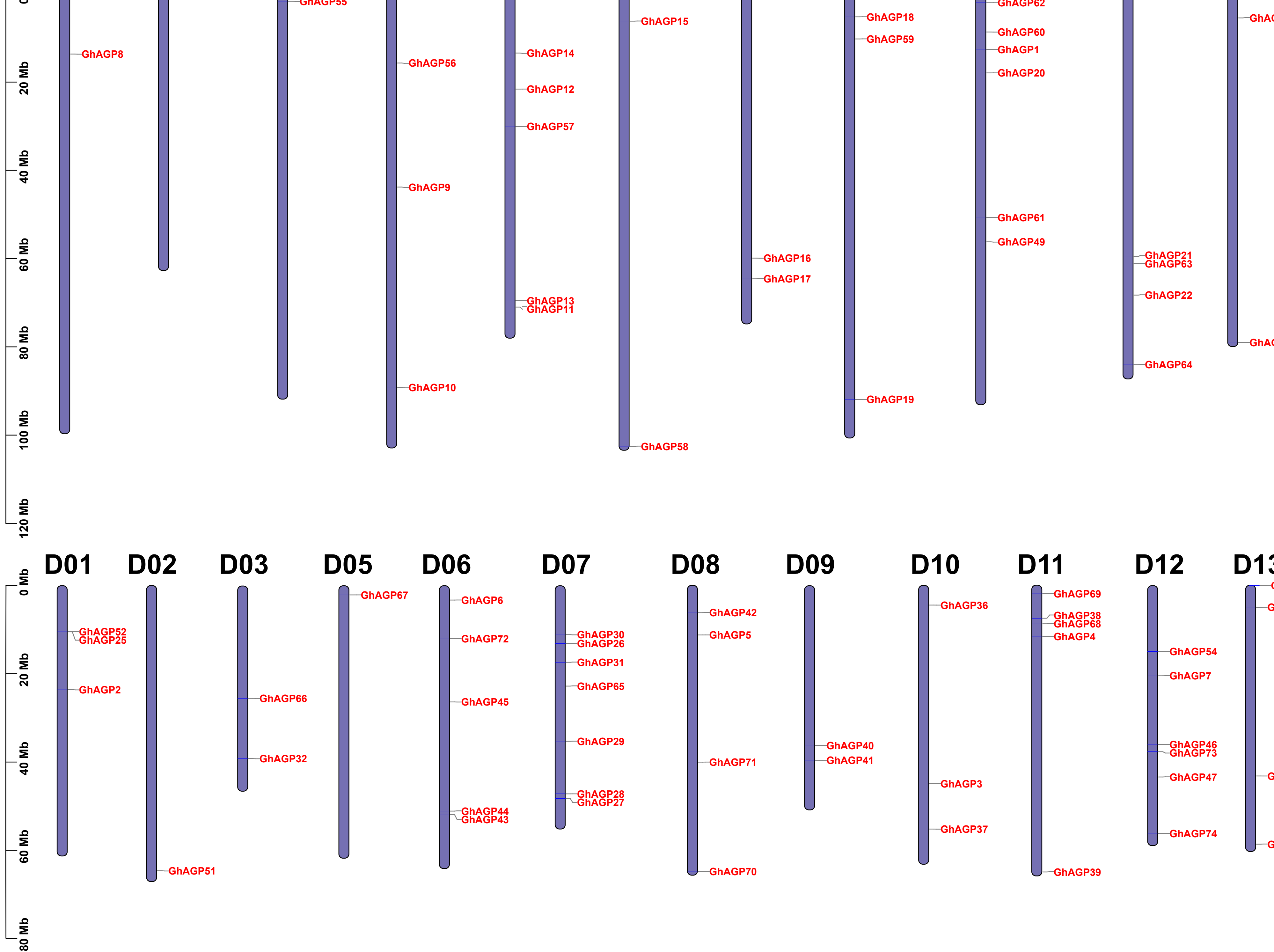

D

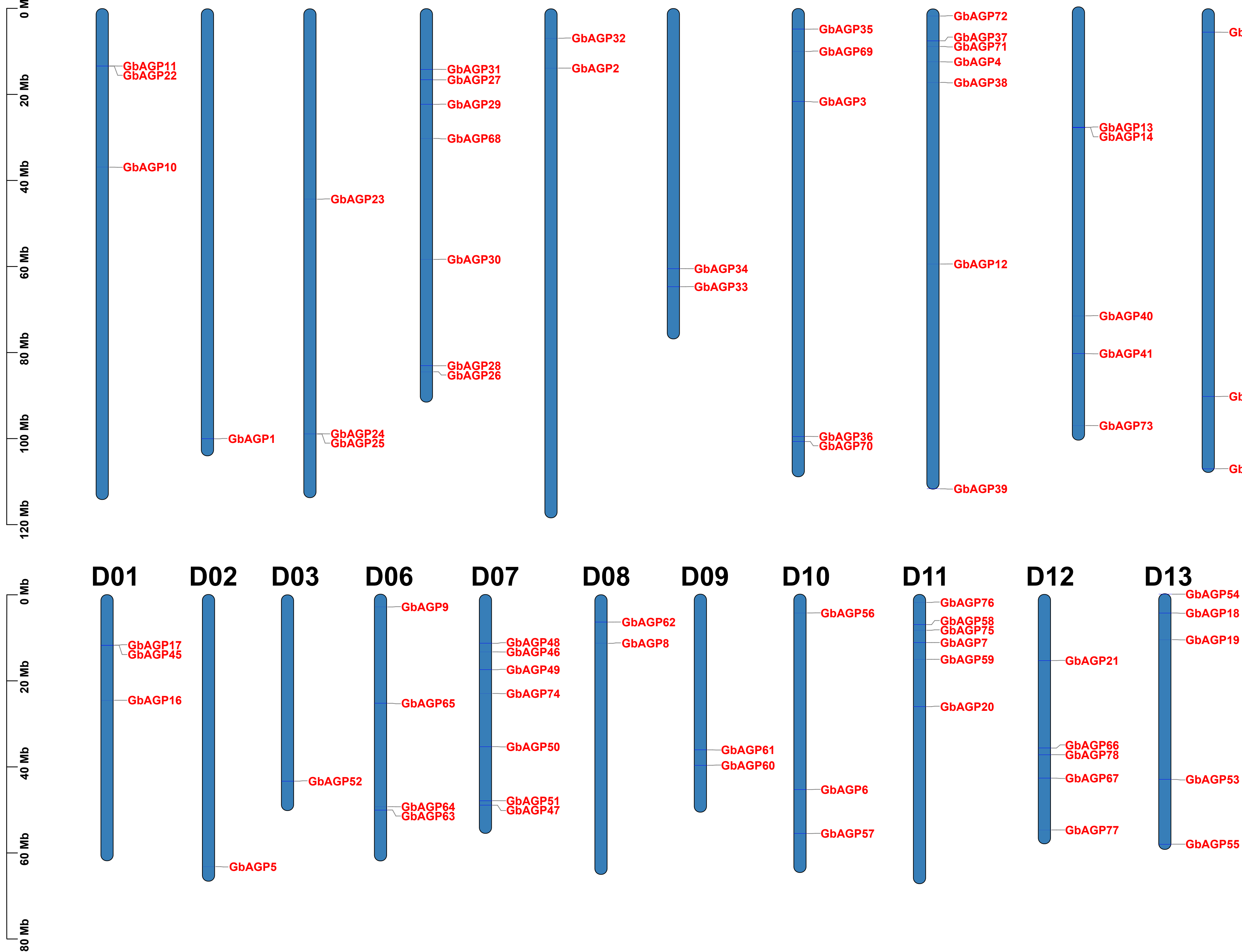

*G. arboreum*  *G. raimondii*  *G. hirsutum*  *G. barbadense*

Supplement: Supplementary file 1 [file ijms-26-04159-s001.zip › Supplymentaty Figure/Figure S5.pdf]

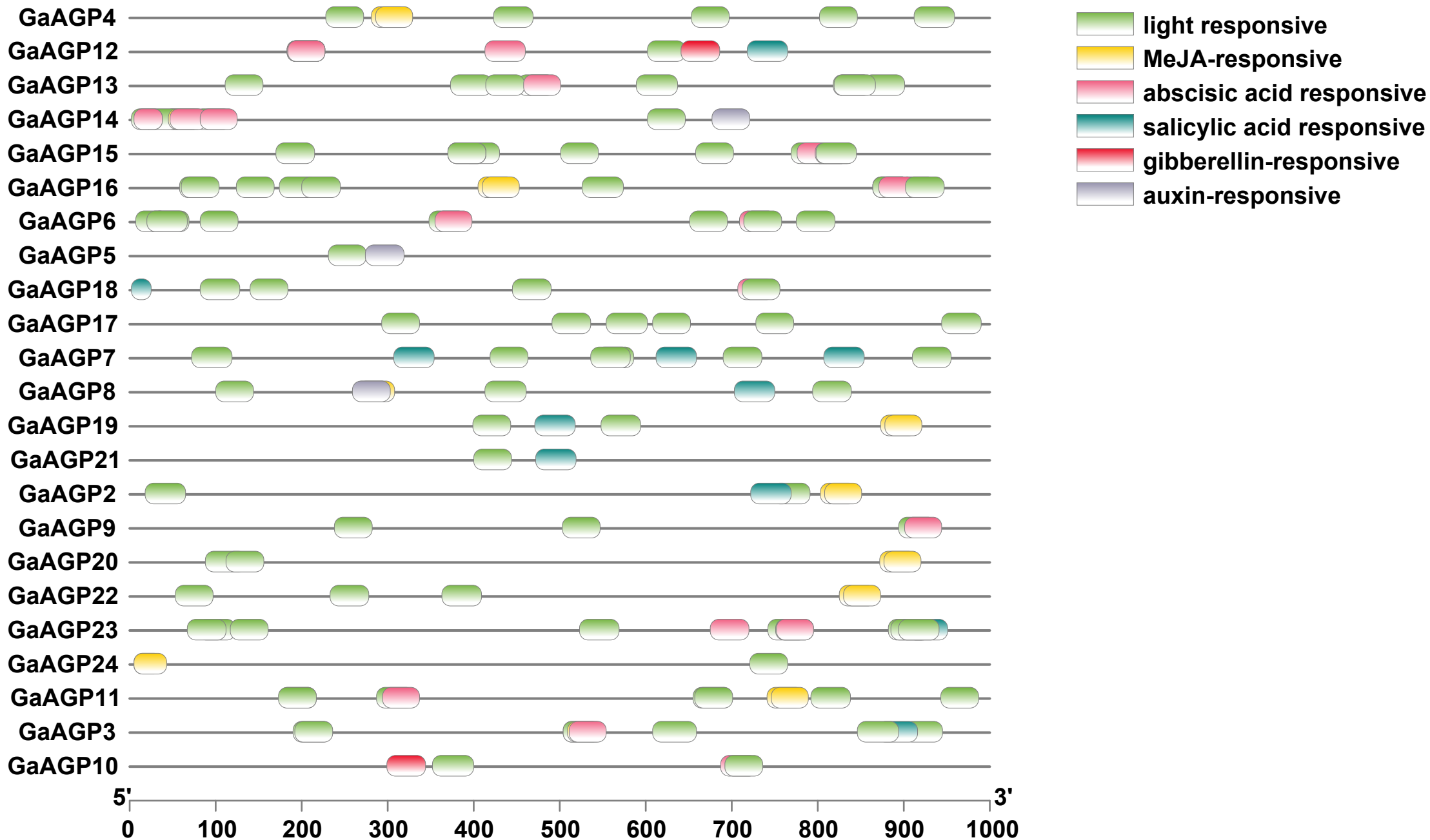

Supplement: Supplementary file 1 [file ijms-26-04159-s001.zip › Supplymentaty Figure/Figure S6a.pdf]

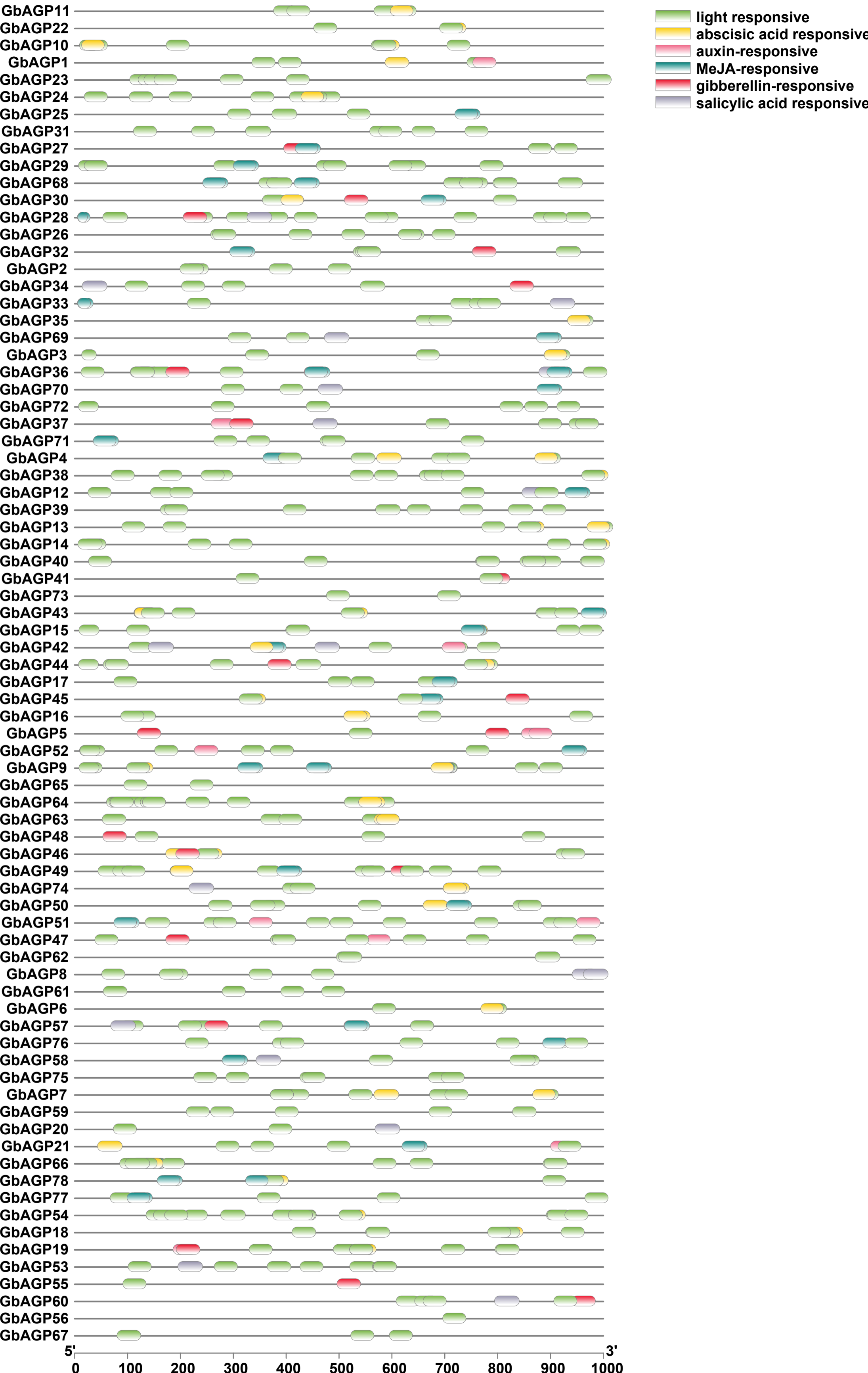

Supplement: Supplementary file 1 [file ijms-26-04159-s001.zip › Supplymentaty Figure/Figure S6b.pdf]

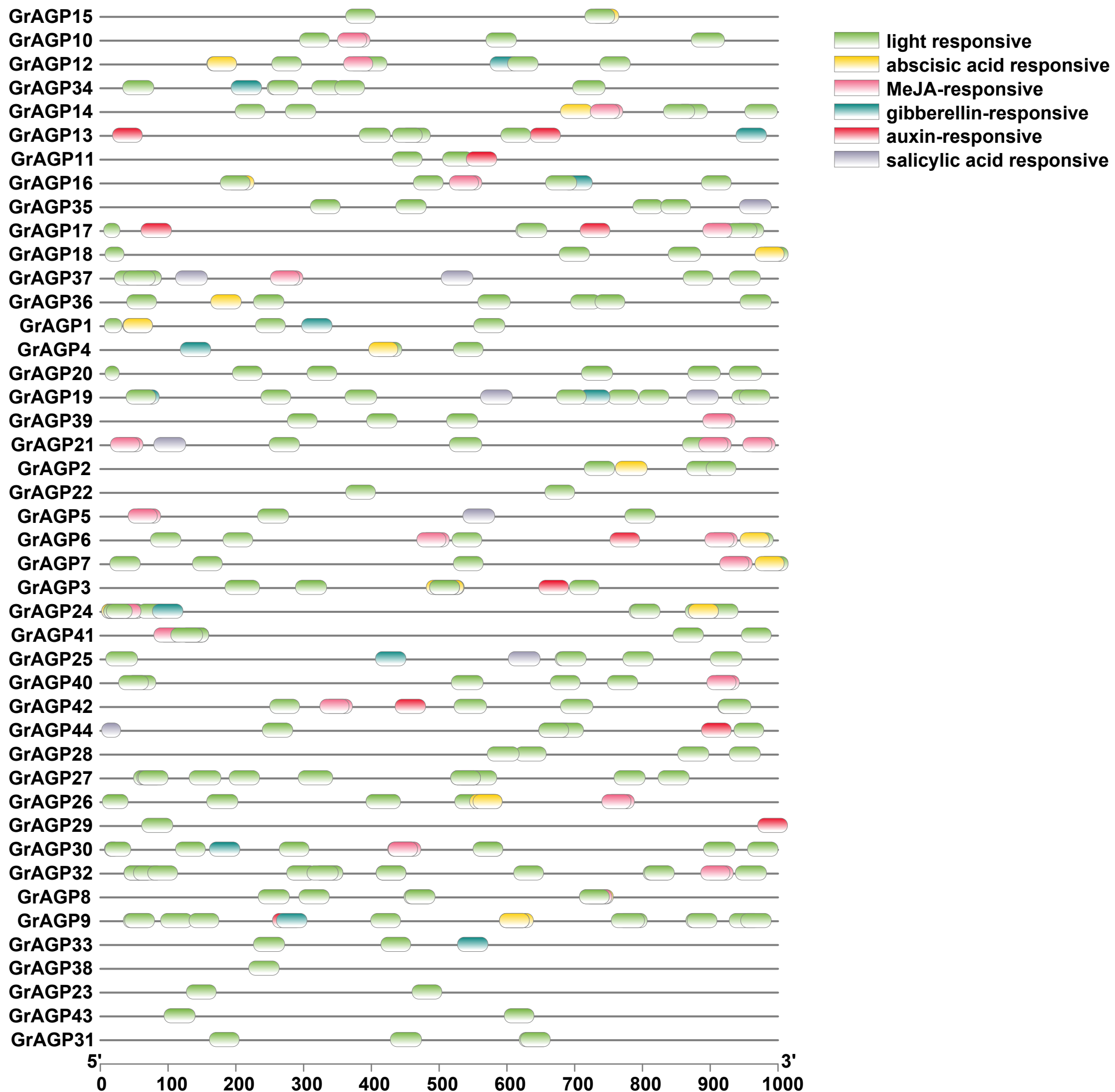

Supplement: Supplementary file 1 [file ijms-26-04159-s001.zip › Supplymentaty Figure/Figure S6c.pdf]

Fluorescence

Bright-field

Merged

35S-GFP

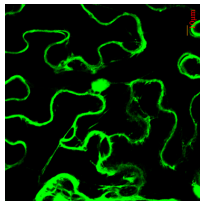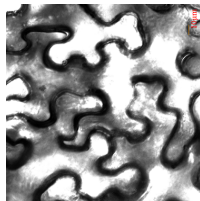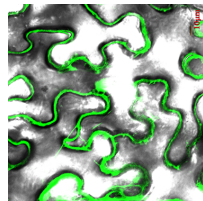

GhAGP50-GFP

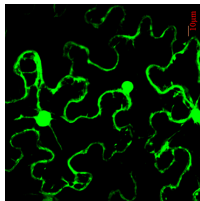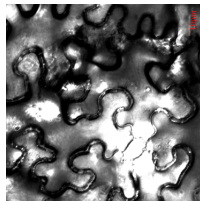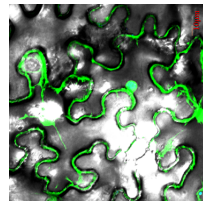

Supplement: Supplementary file 1 [file ijms-26-04159-s001.zip › Supplymentaty Figure/Figure S7.pdf]

# OE-GhAGP50

WT

Line1

Line5

Line11

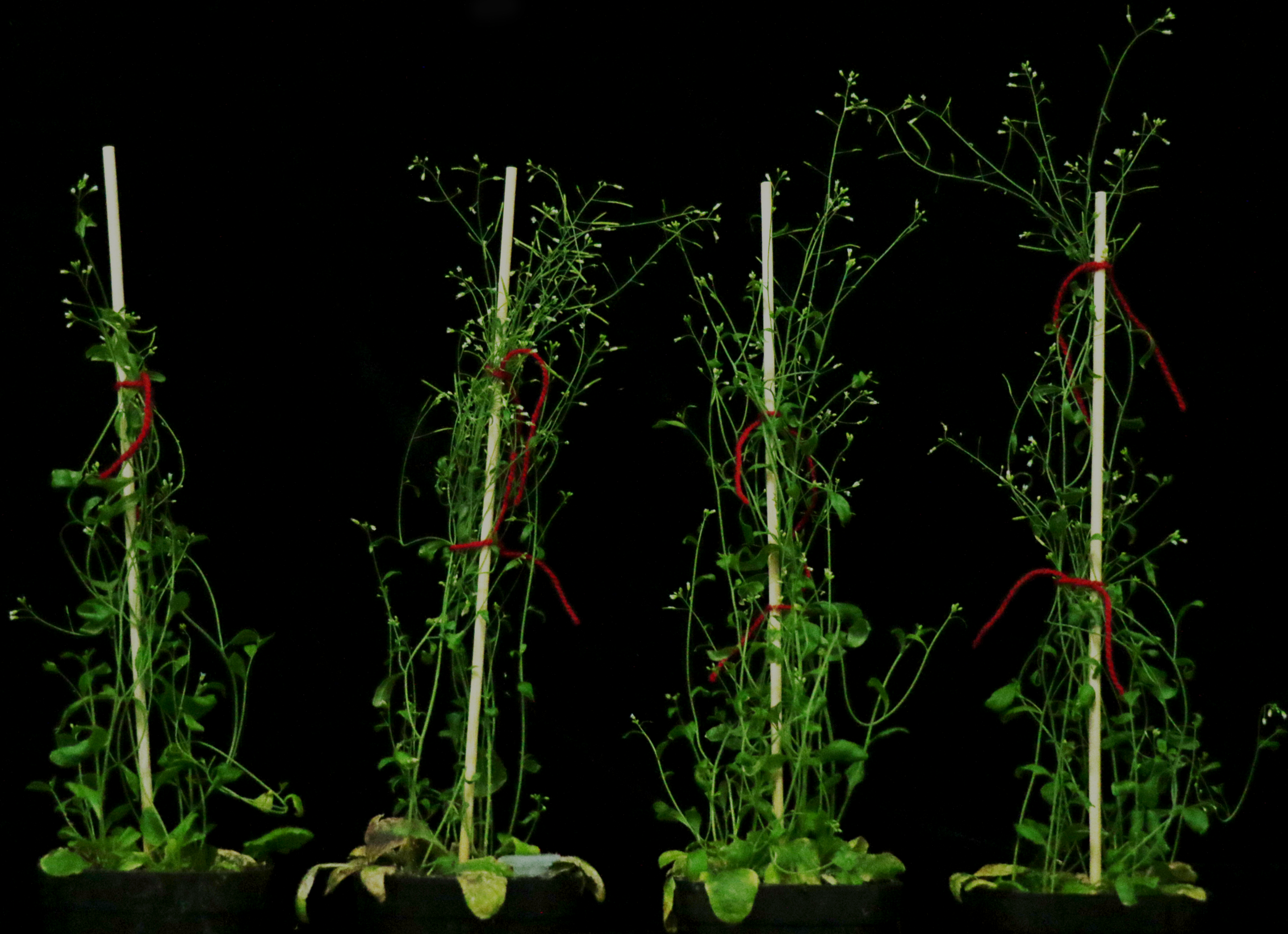

1cm

Supplement: Supplementary file 1 [file ijms-26-04159-s001.zip › Supplymentaty Figure/Figure S8.pdf]
